# Photoactivated organic phosphorescence by stereo-hindrance engineering for mimicking synaptic plasticity

**DOI:** 10.1038/s41377-023-01132-3

**Published:** 2023-04-10

**Authors:** He Wang, Yuan Zhang, Chifeng Zhou, Xiao Wang, Huili Ma, Jun Yin, Huifang Shi, Zhongfu An, Wei Huang

**Affiliations:** 1 Key Laboratory of Flexible Electronics (KLoFE) & Institute of Advanced Materials (IAM), Nanjing Tech University, Nanjing, 211800 China; 2 The Institute of Flexible Electronics (IFE, Future Technologies), Xiamen University, Xiamen, 361005 China; 3 Department of Applied Physics, The Hong Kong Polytechnic University, Kowloon, 999077 Hong Kong China; 4 State Key Laboratory of Organic Electronics and Information Displays & Institute of Advanced Materials (IAM), Nanjing University of Posts & Telecommunications, 9 Wenyuan Road, Nanjing, 210023 China; 5 Frontiers Science Center for Flexible Electronics (FSCFE), MIIT Key Laboratory of Flexible Electronics (KLoFE), Northwestern Polytechnical University, Xi’an, 710072 China

**Keywords:** Optical materials and structures, Applied optics

## Abstract

Purely organic phosphorescent materials with dynamically tunable optical properties and persistent luminescent characteristics enable more novel applications in intelligent optoelectronics. Herein, we reported a concise and universal strategy to achieve photoactivated ultralong phosphorescence at room temperature through stereo-hindrance engineering. Such dynamically photoactivated phosphorescence behavior was ascribed to the suppression of non-radiative transitions and improvement of spin-orbit coupling (SOC) as the variation of the distorted molecular conformation by the synergistic effect of electrostatic repulsion and steric hindrance. This “trainable” phosphorescent behavior was first proposed to mimic biological synaptic plasticity, especially for unique experience-dependent plasticity, by the manipulation of pulse intensity and numbers. This study not only outlines a principle to design newly dynamic phosphorescent materials, but also broadens their utility in intelligent sensors and robotics.

## Abstract (web summary)

Photoactivated organic phosphorescence was successfully achieved by stereo-hindrance engineering, which was first used to mimic multifunctional biological synaptic plasticity and human vision memory.

## Graphical abstract

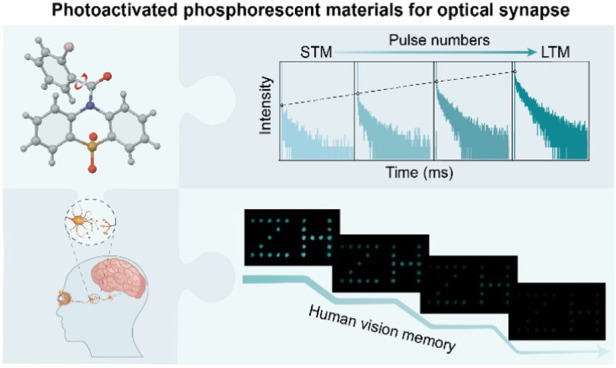

## Introduction

Ultralong organic phosphorescence (UOP) is a unique persistent luminescent behavior of organic materials. It lasts for a period of time after the stoppage of excitation, receiving considerable attention in optoelectronics and bioelectronics recently^1–3^. For a long time, researches related to phosphorescence are mainly based on metal-containing complexes^4,5^. For purely organic molecules, it is a formidable challenge to obtain ultralong phosphorescence at room temperature owing to weak spin-orbit coupling (SOC) between singlet and triplet states as well as serious dissipation of triplet excitons by non-radiative transitions^6–8^. In past years, enormous efforts have been devoted to boosting UOP by stabilization of the triplet excitons at room temperature, including the introduction of heavy atoms^9,10^ or aromatic carbonyls^11,12^ for enhancing SOC and the construction of rigid molecular environment for suppressing non-radiative transitions by crystalline engineering^13–15^, polymerization^16,17^, host-guest doping^18,19^, supramolecular self-assembly^20,21^, etc. With the development of the UOP materials, a series of potential applications were demonstrated, such as anti-counterfeiting and encryption^22–24^, biological imaging^25,26^, chemical sensing^27,28^, and so on. Meanwhile, a series of UOP materials with dynamic behaviors was developed^29–31^. The phosphorescent intensity, lifetimes, colors*,* etc., can be controllably tuned by external stimuli, demonstrating more potential in practical applications, including colorful displays^32,33^, multi-dimensional encryption^34,35^, programmable tags^36,37^, and so forth. So far, it is still of significance to develop new materials with such dynamic phosphorescent behaviors for expanding potential applications in optoelectronics.

**Fig. 1:**
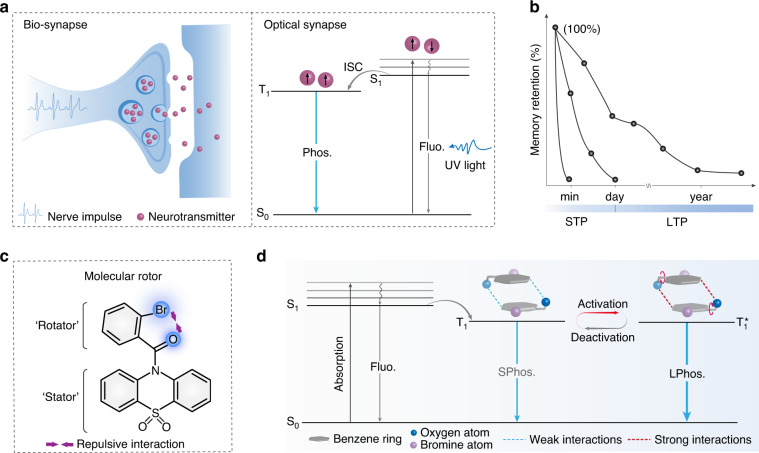
Schematic representation for all-photon synapses plasticity enabled by organic UOP phosphors. **a** Schematic diagram of the activity of biological synapse and the excitation and emission process of phosphorescence. **b** Ebbinghaus forgetting curves. **c** Molecular design for photoactivated organic phosphorescence. **d** Proposed mechanism for photoactivated organic phosphorescence

Synapses are the sites of functional connections between nerve cells, playing a key role in information transmission. Mimicking synaptic functions, including short-term plasticity, long-term plasticity, and experience-dependent plasticity (EDP), etc., is fundamental for the implementation of new “neuromorphic computing” architectures^38–40^. We noted that the excitation and slow decay process of phosphorescence is similar to the biological principle of synapses, in which the neurotransmitters were slowly released from synaptosomes when they received a nerve impulse, causing transient or persistent changes in the postsynaptic membrane (Fig. 1a). Traditional UOP materials can simulate short-term potentiation (STP) and long-term potentiation (LTP) of synaptic plasticity by regulating the excitation intensity and time. But EDP mainly acts on impulses based on past experience, in which the stimulation strength would be reduced as the frequency of events increases. It is necessary to develop material with the ability to build a memory repository based on past-learned events^41^. Photoactivated phosphorescent (PAP) materials, as one type of dynamic UOP materials, can be activated to a state of high phosphorescent intensity and a long-lived lifetime when stimulated by light. Moreover, the molecule can remain in the activated state for a period of time when stopping the stimulation, instead of returning to the original state immediately. So, they can recover to the maximum phosphorescent signal with a relatively small stimulated strength based on the first excitation, which resembles the Hermann Ebbinghaus described the learning-forgetting law in the forgetting curve (Fig. 1b). However, to the best of our knowledge, it is not yet reported a PAP material for the potential application of mimicking synaptic plasticity.

Molecule rotors have the potential for controllably tuning phosphorescence through subtle changes in molecular conformation^42–44^. Inspired by this, we employed a bulky oxidized phenothiazine group with folded conformation as a stator. Abundant oxygen atoms with lone pairs of electrons are in favor of increasing the composition of n orbitals to promote intersystem crossing (ISC) based on El-Sayed rules. A smaller benzoyl group as a rotator is modified on the nitrogen atom of phenothiazine to form an amide bond. While the halogen atom is introduced in the ortho of the benzoyl group, steric hindrance and electrostatic repulsive effect is formed with the neighboring carbonyl unit, which drives the distortion of molecular conformation for generating PAP behavior (Fig. 1c). Herein, both phosphorescent intensity and lifetime of the obtained molecular rotor displayed significant change under the stimulation of UV light. It is mainly attributed to the enhancement of intermolecular interactions following the adjustment of molecular conformation, which facilitated the formation of a more stable excited triplet state (T_1_^*^) with a longer lifetime (Fig. 1d). Accordingly, we categorized the lowest excited triplet state as short phosphorescence (SPhos.) and long phosphorescence (LPhos.), respectively, according to whether the phosphorescence lifetime is lower than 100 ms. Remarkably, the photoactivated organic phosphors could perform multifunctional synaptic plasticity under different pulse intensities and numbers, simultaneously exhibiting great potential in mimicking human visual memory.

## Results

### Synthesis and characterizations

As a proof of concept, we synthesized a series of aromatic amide derivatives, (2-chlorophenyl) (5,5-dioxido-10H-phenothiazin-10-yl)methanone (*o*CDO), (2-bromophenyl) (5,5-dioxido-10H-phenothiazin-10-yl)methanone (*o*BDO), (4-bromophenyl)(5,5-dioxido-10H-phenothiazin-10-yl)methanone (*p*BDO) through two-step reactions (Scheme S1). The chemical structures and purity of the three compounds were thoroughly characterized by ^1^H and ^13^C NMR spectroscopy (Figs. S1–6), elemental analysis, high-performance liquid chromatography (Fig. S7), and single-crystal X-ray diffraction. Moreover, after UV-light irradiation for 5 min, there is no new peak and chemical shifts in the ^1^H and ^13^C NMR spectra, indicating the chemical stability of materials under UV-light irradiation. Notably, there are strong intermolecular interactions and bulky steric hindrance around the oxidized phenothiazine, while a certain degree of freedom and low packing density on the phenyl unit in *o*CDO, *o*BDO, and *p*BDO crystals (Fig. S8), enabling the phosphors with dynamic phosphorescence performance by controllably tuning the molecular motion with stimulation of light.

### Photophysical properties in solution and solid state

The photophysical properties of these organic compounds were firstly investigated in solution. As illustrated in Figure S9a, the absorption spectra in the toluene solution (5.0 × 10^−5^ M) of *o*CDO and *o*BDO were similar to the absorption bands at around 300 nm. While the absorption bands of *p*BDO were redshifted to 304 nm. Their steady-state photoluminescence (PL) spectra of the three compounds in solution displayed semblable profiles with structureless emission bands at 350 and 510 nm (Fig. S9b). Notably, their PL and phosphorescent spectra overlapped well in a dilute solution at 77 K, demonstrating that there exist efficient ISC processes between the excited singlet and triplet states for boosting phosphorescence (Fig. S10). Furthermore, the similar emission bands of phosphorescence spectra for the three molecules indicated that different substituents have less effect on the excited triplet states in the single-molecule state.

**Fig. 2:**
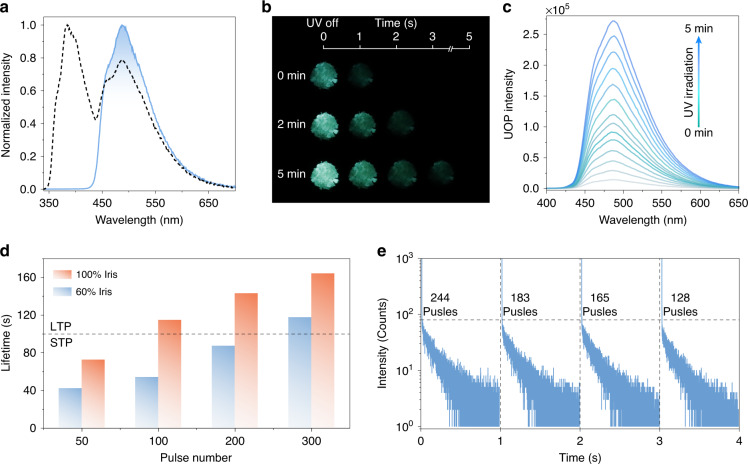
Photophysical properties of the *o*BDO molecules in the solid state under ambient conditions. **a** Steady-state photoluminescence (dotted line) and phosphorescence (solid line) spectra excited by 330 nm. Note that the phosphorescence was collected with a delay time of 8 ms. **b** Afterglow photographs of the *o*BDO crystal under the irradiation of 365 nm UV light for 10 s, 3 and 5 min, respectively. **c** Phosphorescence spectra under the irradiation of 330 nm UV light for 0 to 5 min. The light source power is 284 μW cm^−2^. **d** Phosphorescent lifetimes under the irradiation of pulsed light with different light fluxes (60 and 100% iris) and pulse numbers (50, 100, 200, and 300 pulses). **e** Experience-dependent plasticity enabled by repeatedly exciting the photoactivated organic phosphor at 1 Hz with the light flux of 100% iris. The required pulse numbers to reach the set luminescent intensity were listed alongside the lifetime decay curves

**Fig. 3:**
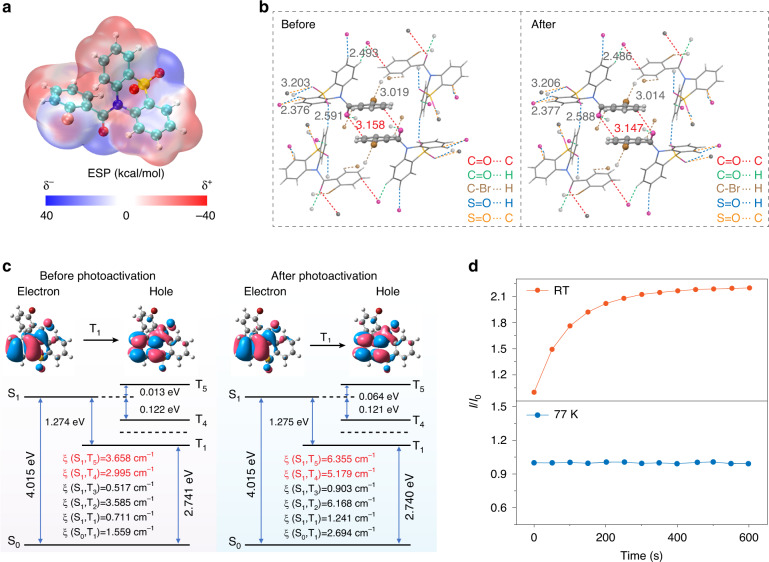
A feasible mechanism for photoactivated organic phosphorescence. **a** Electrostatic potential (ESP) distribution for the *o*BDO molecule in a single molecular state. **b** Intermolecular interactions of the *o*BDO molecule in crystal before and after UV light irradiation. **c** Natural transition orbitals (NTOs) for the lowest excited triplet state and the calculated spin-orbital coupling (SOC) of the *o*BDO molecule before and after photoactivation. **d** Excitation time-dependent phosphorescence intensity variation at room temperature and 77 K, respectively. The light source power is 284 μW cm^−2^

In the solid state, *o*BDO was selected as a model molecule to investigate the photophysical properties. As shown in Fig. 2a, the PL spectrum of *o*BDO showed dual emission bands at 382 and 487 nm with fine vibrational structures under ambient conditions. The higher energy emission at 382 nm can be attributed to fluorescence, according to a short lifetime of 2.18 ns (Fig. S11a). The phosphorescence spectrum with a completely identical emission band at around 487 nm was achieved after a delay time of 8 ms (Fig. 3a). When removed the 365 nm UV light, we observed green afterglow lasting for 1 s in the *o*BDO solid. Remarkably, under the continuous irradiation of 365 nm UV light, a brighter and longer green afterglow was captured by the naked eye for the *o*BDO solid, manifesting obvious PAP behavior (Fig. 2b and SV1). Likewise, the phosphorescence intensity of the *o*BDO exhibited a dramatic increment under the continuous irradiation of 330 nm UV light for 5 min, as illustrated in Fig. 2c. The photoactivated phosphorescent lifetime can reach 187.01 ms, which verified its nature of phosphorescence (Fig. S11b). The time-phosphorescence mapping displayed that the phosphorescence intensity for the *o*BDO phosphor increased rapidly within 10 min and had little change when the excitation time was longer than 15 min (Fig. S12). Notably, the photoactivated phosphorescent behavior is related to the excitation wavelength, which is more sensitive to the short wavelengths in the UV region. It is ascribed to the main absorption of the *o*BDO phosphor in the UV region (Figs. S13, S9a). However, no matter how the excitation wavelength changes, the optimal emission peak of the *o*BDO phosphor remains unchanged (Fig. S14). The influence of irradiation intensity was also investigated by regulating the power density from 5 to 90 μW cm^−2^ (Fig. S15). Remarkably, the PAP behavior could be activated with an ultralow-power density of 5 μW cm^−2^, demonstrating exceptionally low power consumption. When increasing the power density from 10 to 90 μW cm^−2^, the PAP process becomes faster and more obvious. Notably, no matter whether in nitrogen or oxygen, the phosphorescence intensity both displayed obvious enhancement following continuous irradiation of UV light (Fig. S16).

### Simulation of synaptic plasticity

In light of the unique PAP behavior, we further explored the possibility of the *o*BDO phosphor for all-photon synaptic plasticity. We utilized pulsed light (1 Hz) and the excitons to simulate nerve impulses and neurotransmitters in organisms, respectively (Fig. 1a). As the PAP property of the *o*BDO phosphor, pulse intensity and numbers may make a large difference in the generation and deactivation of the excitons, which mimics the performance of synapses under the stimulation of nerve impulses. Firstly, we adjusted different pulse numbers (50, 100, 200, and 300 pulses) under the light flux of 60% iris, and obtained varied lifetime decay curves. It was found that the luminescent intensity and lifetimes were significantly enhanced with the pulse number increase (Fig. S17a and Table S1). When the light flux was adjusted to 100% iris, the phosphorescence intensity and lifetimes were further improved (Fig. S17b and Table S1). Accordingly, we defined the phosphorescence lifetimes of less than 100 ms and intensity of lower than 100 counts as short-term potentiation (STP), oppositely, as long-term potentiation (LTP). As shown in Fig. 2d and S18, all-photon synapse plasticity, including STP, LTP, and the transformation from STP to LTP, can be successfully emulated using the photoactivated phosphor by precisely controlling the pulse intensity and numbers. In addition, we studied the EDP based on such photoactivated organic phosphor. We set the 80 counts as the terminal intensity and tested the lifetime decay curves repeatedly under the pulse light source with 1 Hz and a light flux of 100% iris. The excitation and decay process of phosphorescence is analogous to the learning and forgetting behaviors of humans. As shown in Fig. 2e, when achieving the same luminescence intensity, the required pulse numbers decreased successively in the repeated testing process, which demonstrated that continuous stimulus makes the recovery of memory easier. It is the exact rule of the Ebbinghaus forgetting curves. Therefore, after a memory repository is built on the first excitation, the photoactivated *o*BDO phosphor can display a unique EDP feature by repeated stimulation.

### PAP mechanism investigation

To clarify the unique PAP behavior, theoretical simulation, and single-crystal X-ray diffraction analysis were thoroughly performed. As shown in Fig. 3a, electrostatic potential (δ) analysis of the *o*BDO molecule revealed the carbonyl group and adjacent bromine atom were both distributed with negative δ (δ^-^), which would cause electrostatic repulsion with each other. Besides, the steric hindrance of the bromine atom tends to cause the distortion of phenyl. In the crystal, *o*BDO molecules displayed a head-to-head arrangement, leading to a greater steric hindrance of the bulky oxidized phenothiazine group than that of the benzene ring (Fig. S8). Moreover, the oxidized phenothiazine group was distributed with the strong intermolecular interactions of C=O···H (2.493 Å), S=O···H (2.376 and 2.591 Å), and S=O···C (3.203 Å), which effectively restricted molecular motions for suppressing non-radiative transitions (Fig. 3b). Besides, there are multiple intermolecular interactions of C=O···C (3.158 Å) and C-Br···H (3.019 Å) around benzene rings. Importantly, after photoactivation, the intermolecular interactions between benzene rings were enhanced by shortening the distance of C=O···C from 3.158 to 3.147 Å, which could further facilitate phosphorescence by reducing the non-radiative transitions (Fig. 3b and Tables S2, S3). Next, we further conducted density-functional theory (DFT) calculation to understand the PAP behavior on the basis of the QM/MM model (Fig. S19). From Fig. 3c, it was found that the natural transition orbitals (NTOs) of T_1_ are mainly distributed in the oxidized phenothiazine and carbonyl units. Meanwhile, we also found that the rotation of the benzene ring had less influence on the change of the excited triplet state energy levels before and after UV-light stimulation. It is worth noting that after photoactivation, the SOC of *o*BDO was enhanced by approximately two times based on the molecular conformation before and after UV light irradiation, which is consistent with the experimental result that the phosphorescence intensity is positively related to photoactivation duration (Fig. 2c).

Notably, the torsion angle between the carbonyl group and the benzene ring is 66.72°, suggesting the *o*BDO molecule displays a distorted molecular conformation in crystal (Fig. S20). After photoactivation at room temperature, the torsion angle turns to 66.83°, which proved that the distorted molecular conformation was unstable in its initial state due to the ortho electrostatic repulsion and steric hindrance. At 77 K, the phosphorescence spectrum of *o*BDO exhibited more refined vibration peaks compared to that at room temperature, and the phosphorescent lifetimes were significantly prolonged due to the restricting effect of low temperature on molecular motions (Fig. S21). So, the phosphorescence intensity of *o*BDO was almost invariable under photoactivation for a period of time at 77 K, ascribed to the restricted molecular conformation (Fig. 3d). Taking these results together, we concluded that the phosphorescence was gradually enhanced owing to the suppression of non-radiative transitions and improvement of SOC as the variation of the distorted molecular conformation by a synergistic effect of the electrostatic repulsion and steric hindrance under photoactivation.

### Expansion of PAP molecules

**Fig. 4:**
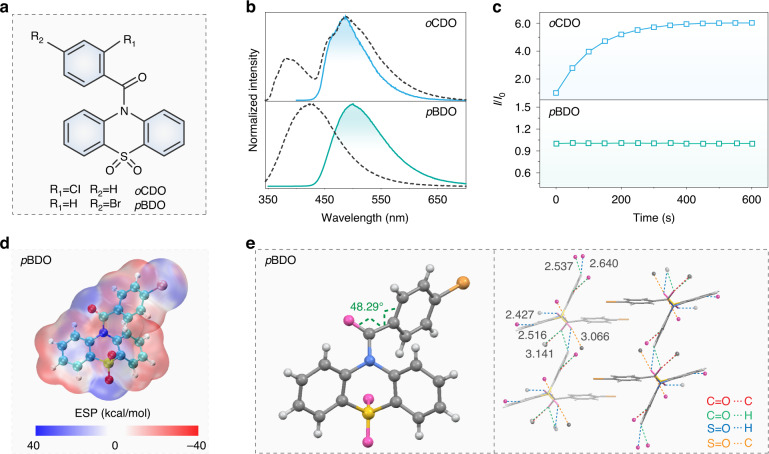
Photophysical properties and crystal structures of *o*CDO and *p*BDO materials. **a** Molecular structures for *o*CDO and *p*BDO. **b** Steady-state photoluminescence (black dotted lines) and phosphorescence (blue and green solid lines) spectra of *o*CDO and *p*BDO crystals under ambient conditions. **c** Excitation time-dependent phosphorescence intensity variation of *o*CDO and *p*BDO crystals. The light source power is 284 μW·cm^−2^. **d** ESP distribution for the *p*BDO molecule in a single molecular state. **e** Molecular torsion angle and intermolecular interactions of *p*BDO molecule in a crystal

To further verify the universality of our design principle for PAP, we studied another two molecules of *o*CDO and *p*BDO (Fig. 4a). As shown in Fig. 4b and Fig. S22, both *o*CDO and *p*BDO exhibited blue steady-state photoluminescence and green long-lived phosphorescence under ambient conditions. At 77 K, they also exhibited refined vibration peaks in the phosphorescence spectra and prolonged phosphorescent lifetimes (Figs. S23, S24). The phosphorescence spectra of *o*CDO and *p*BDO solids showed different degrees of blue shifts at 77 K when compared to that at room temperature, which may be ascribed to the emission of mono-molecular states in a more rigid environment. However, only *o*CDO displayed similar PAP behavior with *o*BDO under the continuous irradiation of UV light in the solid state. There is no change in the phosphorescence intensity of *p*BDO under excitation by UV light (Fig. 4c). From Fig. S25 and Table S4, it is worth noting that the *o*CDO molecule has the same ortho effect of electrostatic repulsion and steric hindrance between carbonyl and chlorine units as the *o*BDO molecule. Whereas for the *p*BDO molecule, the bromine atom was substituted on the para-position of the carbonyl, the effect of electrostatic repulsion and steric hindrance with each other was absent (Fig. 4d). Moreover, the torsion angle between the carbonyl group and benzene ring in *p*BDO molecule (48.29°) is obviously less than that of *o*BDO (66.72°) and *o*CDO (65.63°), convincingly confirming the key role of the ortho effect on the distortion of molecular conformation (Fig. 4e). In addition, the *p*BDO displayed similar head-to-head molecular packing to the *o*BDO in crystal (Fig. S8), but the intermolecular interactions were mainly concentrated on the oxidized phenothiazine. There were no obvious restricted interactions around benzene rings (Fig. 4e). Therefore, the difference in electrostatic and steric hindrance effects and the distribution of intermolecular interactions made the photoactivated behavior of *p*BDO disappear. To sum up, the PAP behavior was closely related to the ortho effects of electrostatic repulsion and steric hindrance.

## Discussion

**Fig. 5:**
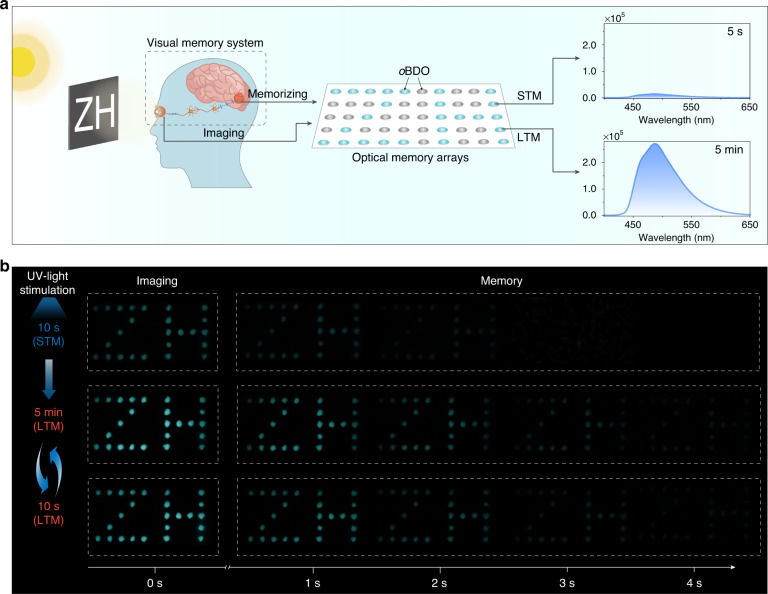
Potential application of the photoactivated organic phosphor for mimicking human visual memory. **a** A schematic diagram of the optical-visual memory arrays used for simulating different visual memory behaviors of STM, LTM, and EDP. **b** Demonstration of multimodal visual memory processes, including the transformation from STM to LTM and experience-dependent memory behavior

Visual system is of important for the human perception of external stimuli, which can be divided into imaging and memory parts. Most prototype devices for image sensing can acquire real-time images, but cannot preserve the relevant information for a while after removing the stimulus^45,46^. Herein, given the PAP behavior of the *o*BDO phosphor, we try to utilize the varied phosphorescent intensity to simulate the imaging quality and the long phosphorescent lifetime to mimic the memory time of the human vision, respectively. As depicted in Fig. 5a, an optical-visual memory array of 5 × 10 pixels was fabricated with *o*BDO phosphor. The letters “ZH” were selected as models of the image information. When stimulated by light for 10 s and 5 min, the photoactivated visual memory array can display short-term memory (STM) and long-term memory (LTM) of “ZH” information, respectively. As shown in Fig. 5b, the array showed a blurry “ZH” after stimulation for 10 s, and the information can be only kept for a short time, demonstrating a typical STM characteristic. Impressively, the STM for the “ZH” information can be dynamically converted to LTM mode by continuous stimulation for 5 min. A clearer imaging of the “ZH” information was achieved and kept for a longer time. Importantly, when the optical memory array was in LTM mode, it can easily recall the relevant memory about the past event even if only receiving the stimulation for 10 s, exhibiting unique experience-dependent memory behavior. This optical-visual system possesses the features of visualization, high contrast, and dynamic transformation, exhibiting great potential in mimicking visual memory systems and intelligent robotics.

In summary, we reported a series of organic phosphors with a photoactivated phosphorescence behavior by stereo-hindrance engineering under ambient conditions. The phosphorescent intensity exhibits nearly fourfold increase under the stimulation of UV light. Combined with experimental and theoretical calculations, the photoactivated phosphorescence behavior was ascribed to the suppression of non-radiative transitions and improvement of SOC as the variation of the distorted molecular conformation by the synergistic effect of the electrostatic repulsion and steric hindrance under the irradiation of UV light. Remarkably, the varied phosphorescent intensity and lifetimes under different pulse intensities and numbers could be utilized to emulate the synaptic plasticity and visual memory behavior of humans. We firstly demonstrated the potential application of such a unique phosphorescent behavior for mimicking biological synaptic plasticity, especially for unique experience-dependent plasticity. This finding not only enriches stimuli-responsive organic phosphorescent materials but also expands the scope of the applications of organic phosphorescent materials in smart optoelectronics and multifunctional robotics.

## Materials and methods

### Materials

All reagents and solvents were purchased from commercial sources and used without further treatment. All products were purified by flash column chromatography. The silica gel was 200–300 mesh. And the resulting crystals were obtained using crystallization techniques such as slow evaporation in ethyl acetate at room temperature.

### Synthesis of photoactivated UOP materials

The intermediate aromatic amide derivatives based on phenothiazine were synthesized according to the reported methods^47^.

For the synthesis of (2-chlorophenyl) (5,5-dioxido-10H-phenothiazin-10-yl) methanone (*o*CDO): (2-chlorophenyl) (10H-phenothiazin-10-yl)methanone (2 g, 5.9 mmol) was dissolved in a 100 mL round-bottom flask with acetic acid (15 mL) and H_2_O_2_ (3 mL). The mixture was stirred at 120 °C for 2 h. And then, the reaction mixture was washed with dichloromethane three times. The product was purified by column chromatography using petroleum ether/ethyl acetate (8:1 v/v) as eluent to afford a white powder as the product (1.56 g, 72%). ^1^H NMR (400 MHz, DMSO-*d*_6_) δ 8.06 (d, *J* = 7.6 Hz, 2H), 7.67 (d, *J* = 3.7 Hz, 4H), 7.60 (dd, *J* = 7.6, 4.5 Hz, 3H), 7.46-7.40 (m, 1H), 7.26 (d, *J* = 7.8 Hz, 2H). ^13^C NMR (101 MHz, DMSO-*d*_6_) δ 164.91, 138.34, 134.48, 133.40, 133.37, 131.94, 130.80, 129.98, 128.10, 127.93, 127.37, 126.90, 123.59. Anal. Calculated for C_19_H_12_ClNO_3_S: C, 61.71; H, 3.27; N, 3.79; S, 8.67. Found: C, 61.84; H, 3.10; N, 3.71; S, 8.63.

For the synthesis of (2-bromophenyl) (5,5-dioxido-10H-phenothiazin-10-yl) methanone (*o*BDO): Based on the same synthetic approach for *o*CDO, the reactants of (2-bromophenyl) (10H-phenothiazin-10-yl) methanone was 2 g (5.2 mmol), acetic acid was 15 mL, and H_2_O_2_ was 3 mL. The product was obtained as a white powder (1.47 g, 68%).^1^H NMR (400 MHz, DMSO-*d*_6_) δ 8.09-8.03 (m, 2H), 7.78 (dd, *J* = 8.0, 1.1 Hz, 1H), 7.71-7.57 (m, 6H), 7.38-7.24 (m, 2H), 7.14 (dd, *J* = 7.6, 1.8 Hz, 1H). ^13^C NMR (101 MHz, DMSO-*d*_6_) δ 165.66, 138.42, 136.44, 133.54, 133.44, 133.22, 132.04, 128.19, 127.84, 127.74, 126.96, 123.65, 120.23. Anal. Calculated for C_19_H_12_BrNO_3_S: C, 55.09; H, 2.92; N, 3.38; S, 7.74. Found: C, 55.22; H, 2.51; N, 3.30; S, 7.70.

For the synthesis of (4-bromophenyl) (5,5-dioxido-10H-phenothiazin-10-yl) methanone (*p*BDO): Based on the same synthetic approach for *o*CDO, the reactants of (4-bromophenyl) (10H-phenothiazin-10-yl) methanone was 2 g (5.2 mmol), acetic acid was 15 mL, and H_2_O_2_ was 3 mL. The product was obtained as a white powder (1.68 g, 77%).^1^H NMR (400 MHz, DMSO-*d*_6_) δ 8.07 (d, *J* = 7.7 Hz, 2H), 7.85 (d, *J* = 8.2 Hz, 2H), 7.67 (t, *J* = 7.8 Hz, 2H), 7.59 (t, *J* = 8.4 Hz, 4H), 7.40 (d, *J* = 8.5 Hz, 2H). ^13^C NMR (101 MHz, DMSO-*d*_6_) δ 166.83, 139.31, 133.92, 133.35, 132.61, 131.57, 130.49, 127.65, 127.28, 125.00, 123.40. Anal. Calculated for C_19_H_12_BrNO_3_S: C, 55.09; H, 2.92; N, 3.38; S, 7.74. Found: C, 55.11; H, 3.02; N, 3.27; S, 7.76.

### Physical characterization

Nuclear magnetic resonance (^1^H NMR and ^13^C NMR) spectra were obtained on a Bruker Ultra Shield Plus 400 MHz spectrometer. Deuterium dimethyl sulfoxide was as a solvent and the chemical shift was calibrated using tetramethylsilane (TMS) as the internal standard. Resonance patterns were recorded with the notation s (singlet), d (double), t (triplet), q (quartet), and m (multiplet). High-performance liquid chromatography (HPLC) was performed using a SunFireTM C18 column conjugated to an ACQUITY UPLCH-class water HPLC system. Steady-state photoluminescence, phosphorescence, and excitation time-dependent phosphorescence emission spectra were measured using Hitachi F7100. The lifetime decay curves under different light fluxes and pulse numbers were obtained on an Edinburgh FLSP1000 fluorescence spectrophotometer equipped with a nanosecond hydrogen flash-lamp (nF920) and a microsecond flash-lamp (μF900), respectively. The luminescent photos were taken by a Cannon EOS 700D camera under the irradiation of a hand-held UV lamp (365 nm). X-ray crystallography was achieved using a Bruker SMART APEX-II CCD diffractometer with graphite monochromated Mo-Kα radiation.

### DFT calculations

The electronic structures of all organic molecules in crystals were performed by using hybrid quantum mechanics/molecular mechanics (QM/MM) theory, including the central QM part and the surrounding MM part modeled by the Universal Force Field (UFF), where a 5 × 5 × 5 supercell was extracted from X-ray diffraction crystal structure. Two-layer ONIOM method^48,49^ was implemented to perform QM/MM calculations using Gaussian 09 package^50^, and the equilibrium geometries of the ground state(S_0_) and the lowest triplet state (T_1_) by (UDFT)B3LYP functional, together with a def-SVP basis set. Based on the optimized T_1_ geometry, the natural transition orbitals (NTOs) of T_1_ state and spin-orbit coupling coefficients (ξ) were calculated at the TD-DFT-B3LYP level. The electrostatic potential (ESP) isosurface was evaluated by using Multiwfn^51,52^.

## Supplementary information

Revised supplementary information

SV1

Authorship Change Form

oBDO-a.cif

oBDO-b.cif

oCDO.cif

pBDO.cif

oBDO-a-check

oBDO-b-check

oCDO-check

pBDO-check
