# Supplementary material for: Photoactivated organic phosphorescence by stereo-hindrance engineering for mimicking synaptic plasticity
Source: Light Sci Appl. 2023 Apr 10;12:90. doi: 10.1038/s41377-023-01132-3 (PMC10086021; doi:10.1038/s41377-023-01132-3)
Supplement: Supplementary file 1 — Revised supplementary information [file 41377_2023_1132_MOESM1_ESM.docx]

Supplementary Information for

**Photoactivated organic phosphorescence by stereo-hindrance engineering for mimicking**

**synaptic plasticity**

He Wang,^1^ Yuan Zhang,^1^ Chifeng Zhou,^1^ Xiao Wang,^2^ Huili Ma,^1^ Jun Yin,^3^ Huifang Shi,^1,4^* Zhongfu An,^1, 2^* Wei Huang^1, 2, 4, 5^*

^1^Key Laboratory of Flexible Electronics (KLoFE) & Institute of Advanced Materials (IAM), Nanjing Tech University, Nanjing 211816, China.

^2^The Institute of Flexible Electronics (IFE, Future Technologies), Xiamen University, Xiamen, 361005, China.

^3^Department of Applied Physics, The Hong Kong Polytechnic University, Kowloon 999077 Hong Kong, China.

^4^State Key Laboratory of Organic Electronics and Information Displays & Institute of Advanced Materials (IAM), Nanjing University of Posts & Telecommunications, 9 Wenyuan Road, Nanjing 210023, China.

^5^Frontiers Science Center for Flexible Electronics (FSCFE), MIIT Key Laboratory of Flexible Electronics (KLoFE), Northwestern Polytechnical University, Xi'an 710072, China.

Correspondence: Huifang Shi (E-mail: iamhfshi@njtech.edu.cn); Zhongfu An (E-mail: iamzfan@njtech.edu.cn); Wei Huang (E-mail: vc@nwpu.edu.cn)

Contents

I. Experimental section

II. Additional photophysical properties of phosphors

III. PAP mechanism investigation and molecular expansion

IV. Supplementary video

**I. Experimental Section**


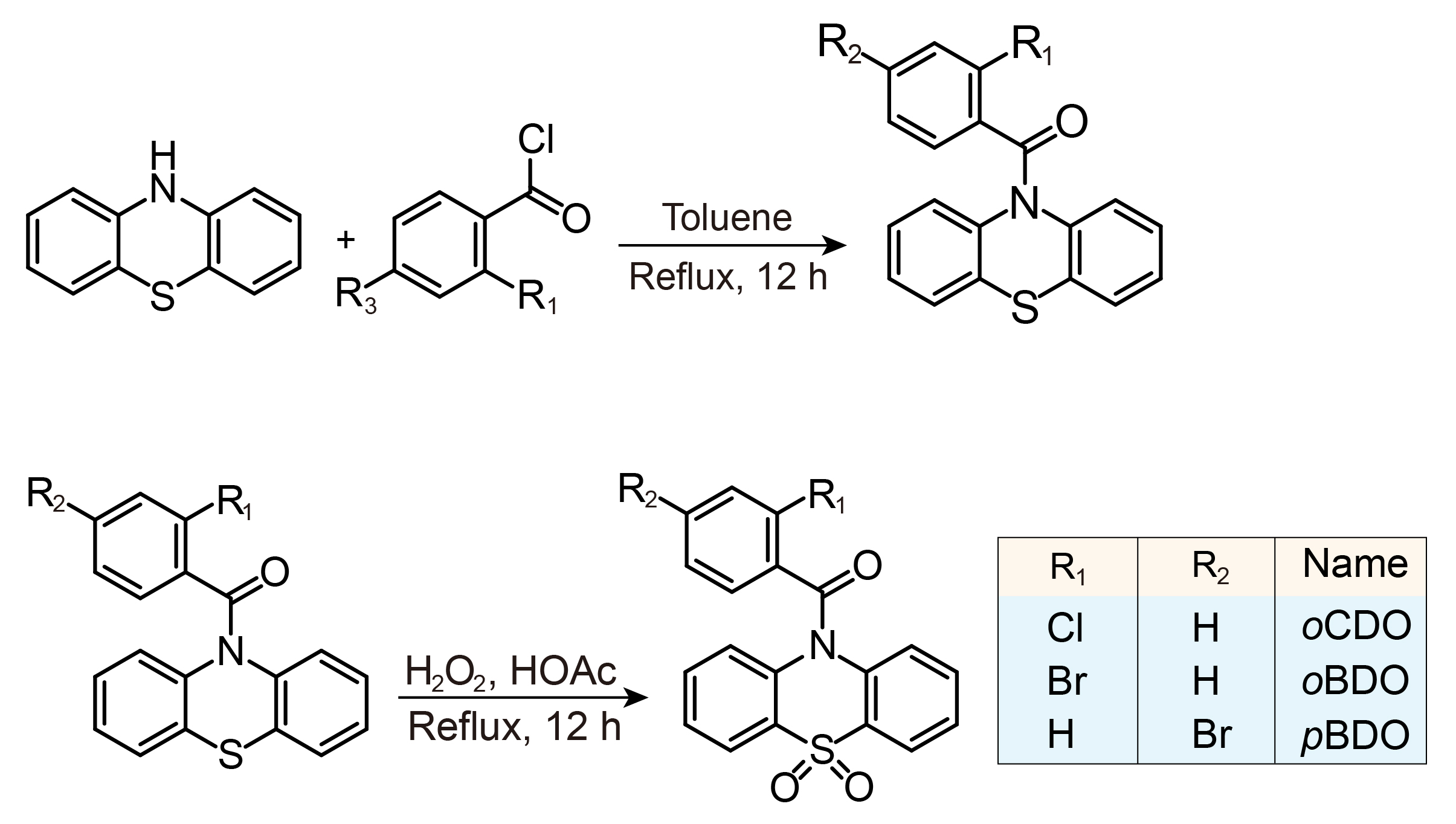


**Scheme 1.** Synthetic routes of *o*CDO, *o*BDO, and *p*BDO molecules.

The ^1^H and ^13^C NMR data of *o*CDO, *o*BDO and *p*BDO molecules after UV-light irradiation for 5 minutes:

For *o*CDO molecule: ^1^H NMR (400 MHz, DMSO-*d*_6_) δ 8.06 (d, *J*=7.7 Hz, 2H), 7.67 (d, *J*=3.7 Hz, 4H), 7.60 (dd, *J*=7.7, 4.5 Hz, 3H), 7.43 (s, 1H), 7.26 (d, *J*=7.1 Hz, 2H). ^13^C NMR (101 MHz, DMSO-*d*_6_) δ 164.93, 138.36, 134.49, 133.45, 133.39, 131.98, 130.80, 130.00, 128.14, 127.95, 127.41, 126.93, 123.62.

For *o*BDO molecule: ^1^H NMR (400 MHz, DMSO-*d*_6_) δ 8.05-8.00 (m, 2H), 7.74 (d, *J*=9.1 Hz, 1H), 7.68-7.53 (m, 6H), 7.34-7.21 (m, 2H), 7.10 (dd, *J*=7.6, 1.8 Hz, 1H). ^13^C NMR (101 MHz, DMSO-*d*_6_) δ 165.65, 138.42, 136.44, 133.54, 133.44, 133.22, 132.04, 128.20, 127.85, 127.74, 126.97, 123.65, 120.23.

For *p*BDO molecule: ^1^H NMR (400 MHz, DMSO-*d*_6_) δ 8.06 (d, *J*=7.5 Hz, 2H), 7.84 (d, *J*=7.9 Hz, 2H), 7.66 (t, *J*=8.1 Hz, 2H), 7.60 (d, *J*=9.1 Hz, 4H), 7.40 (d, *J*=8.0 Hz, 2H). ^13^C NMR (101 MHz, DMSO-*d*_6_) δ 166.82, 139.30, 133.91, 133.35, 132.60, 131.57, 130.49, 127.64, 127.28, 125.00, 123.40.


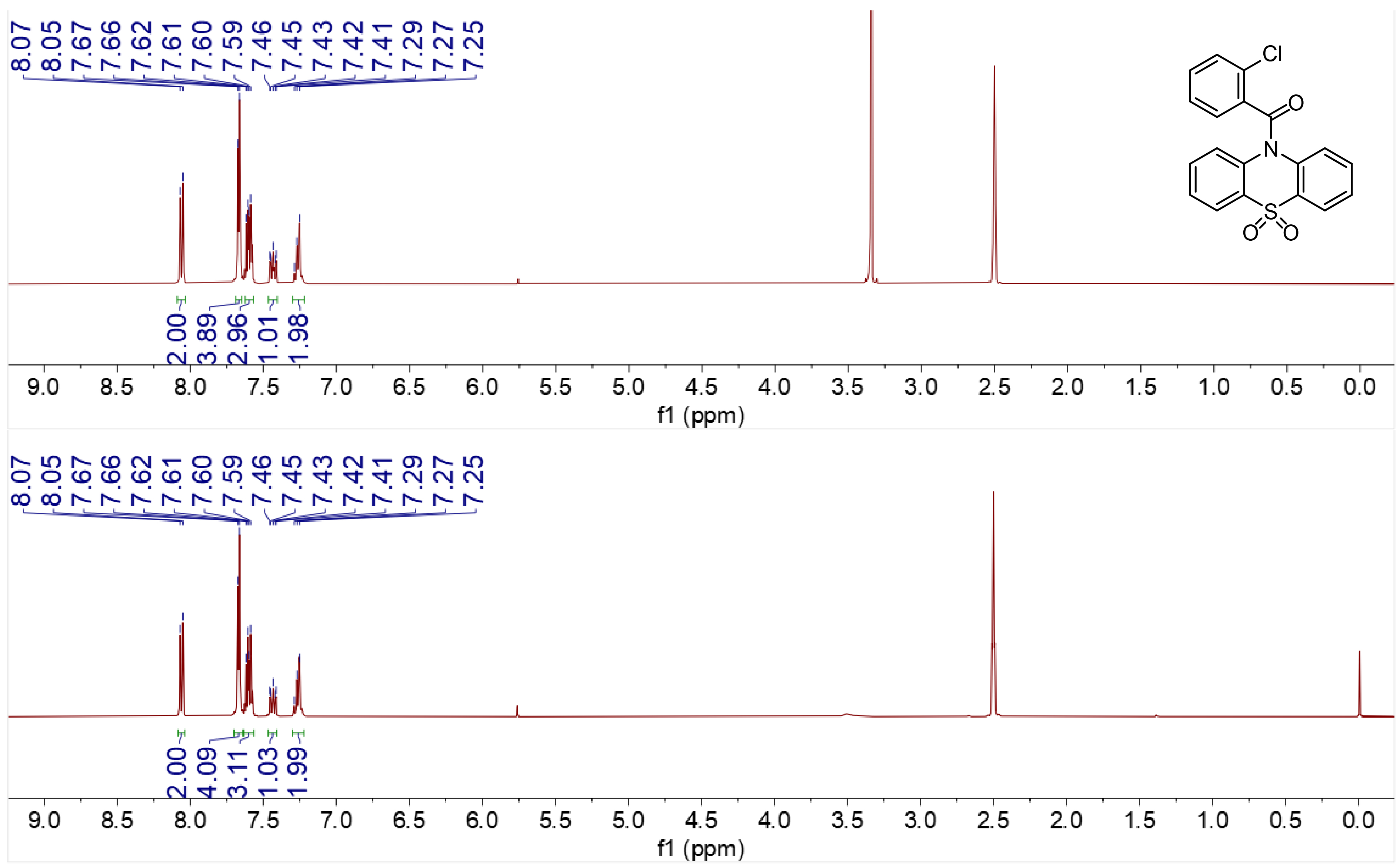


**Figure S1.** ^1^H NMR spectra of *o*CDO molecule before (top) and after (bottom) UV-light irradiation in DMSO-*d*_6_.


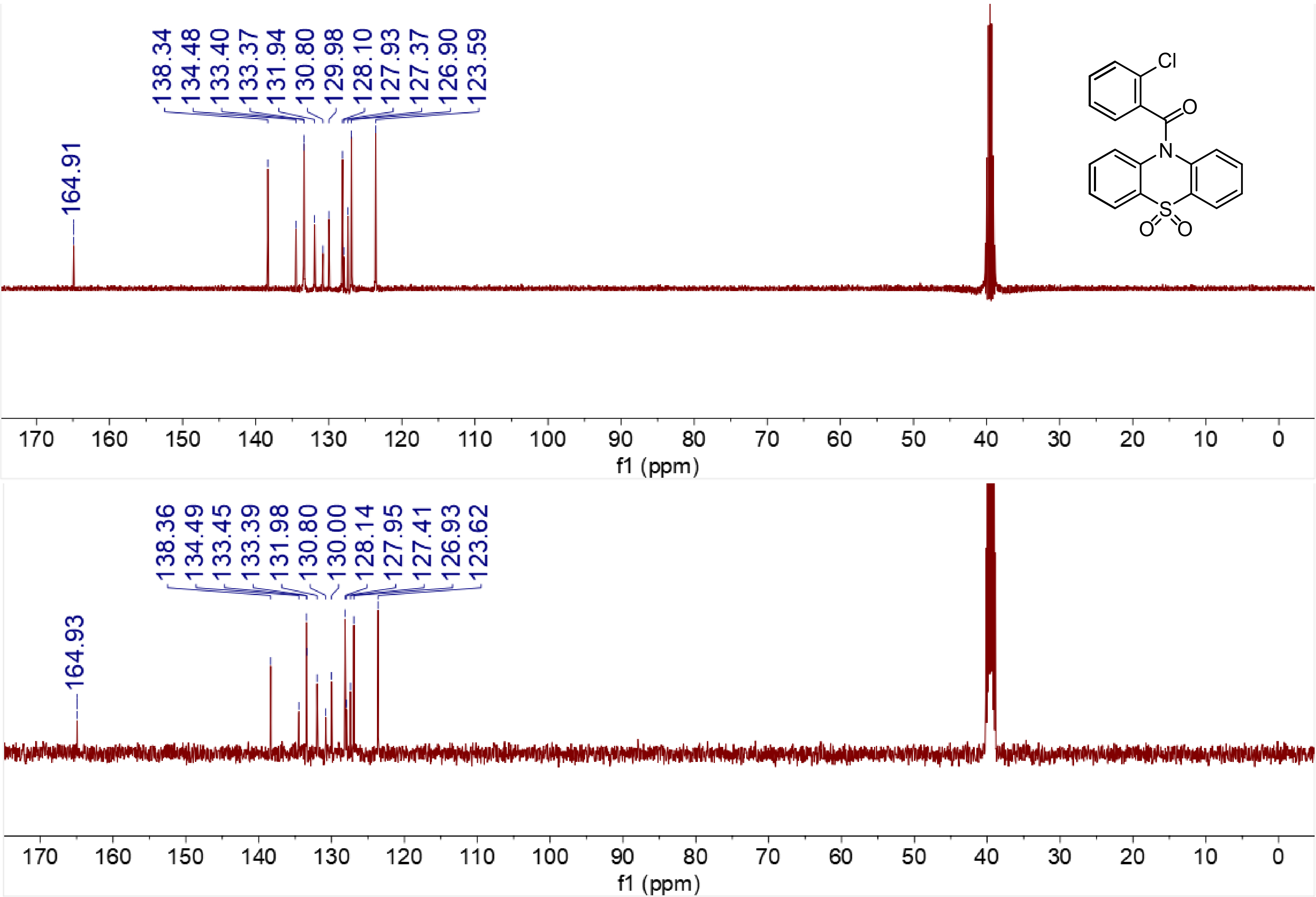


**Figure S2.** ^13^C NMR spectra of *o*CDO molecule before (top) and after (bottom) UV-light irradiation in DMSO-*d*_6_.


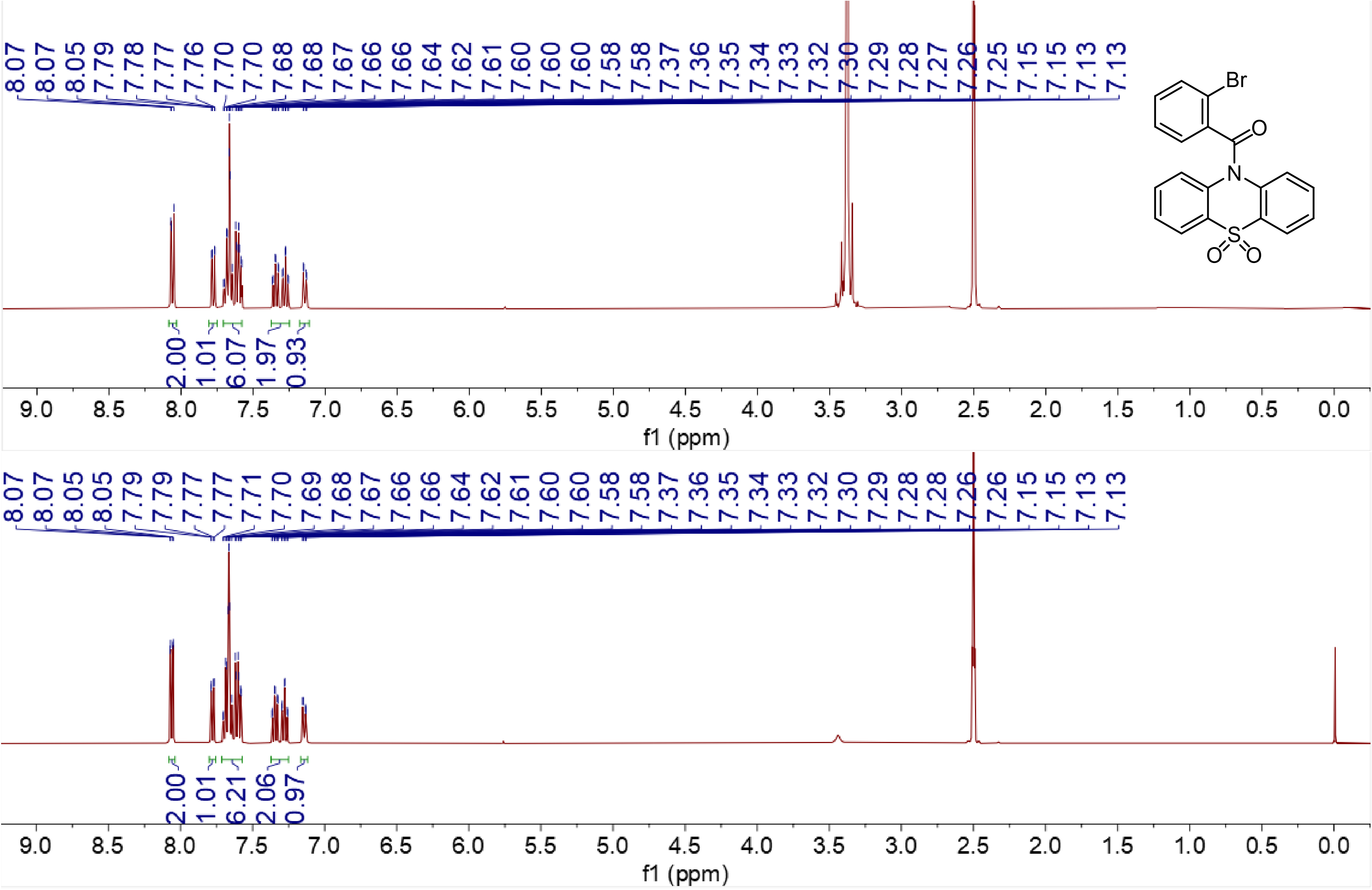


**Figure S3.** ^1^H NMR spectra of *o*BDO molecule before (top) and after (bottom) UV-light irradiation in DMSO-*d*_6_.


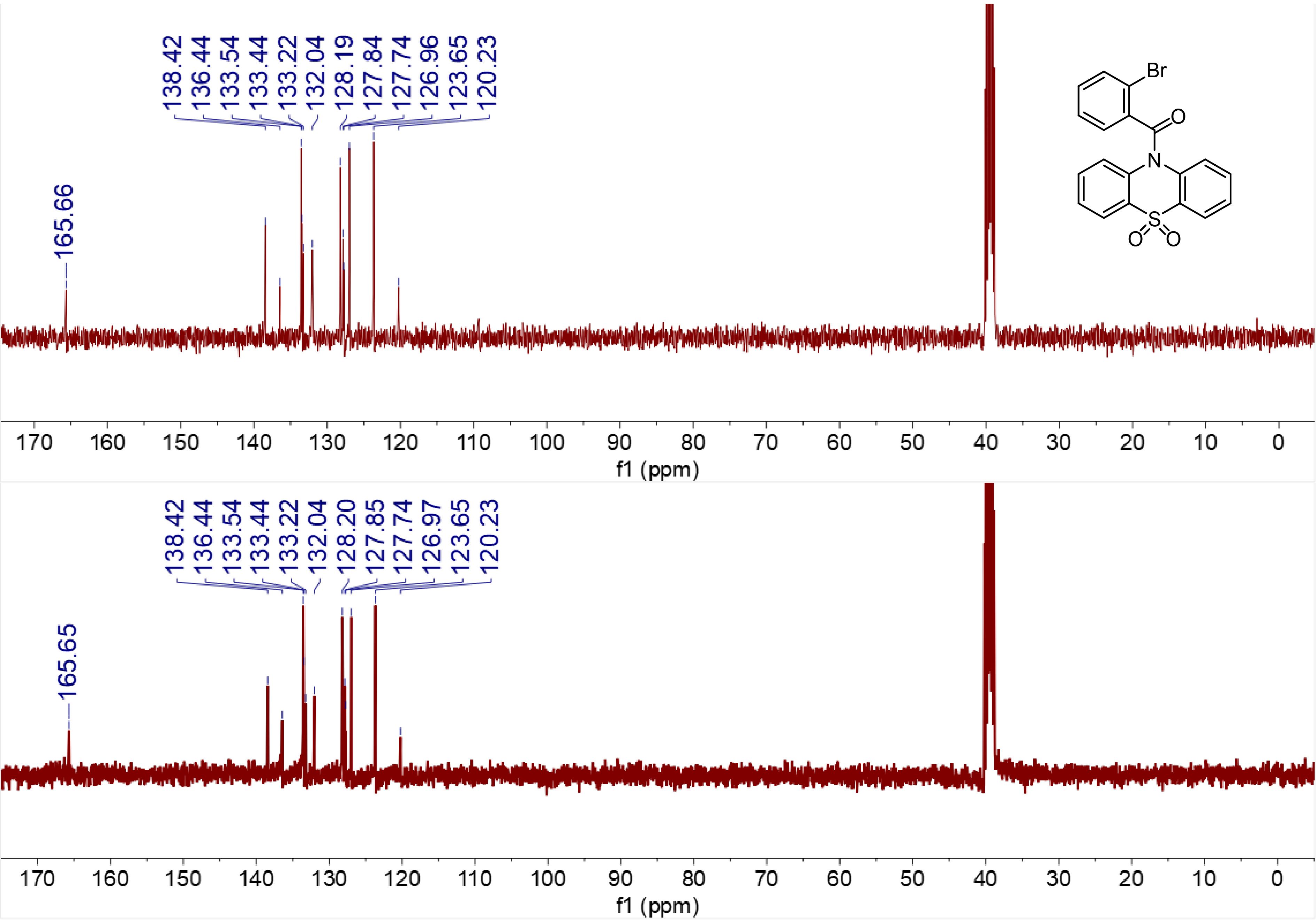


**Figure S4.** ^13^C NMR spectra of *o*BDO molecule before (top) and after (bottom) UV-light irradiation in DMSO-*d*_6_.


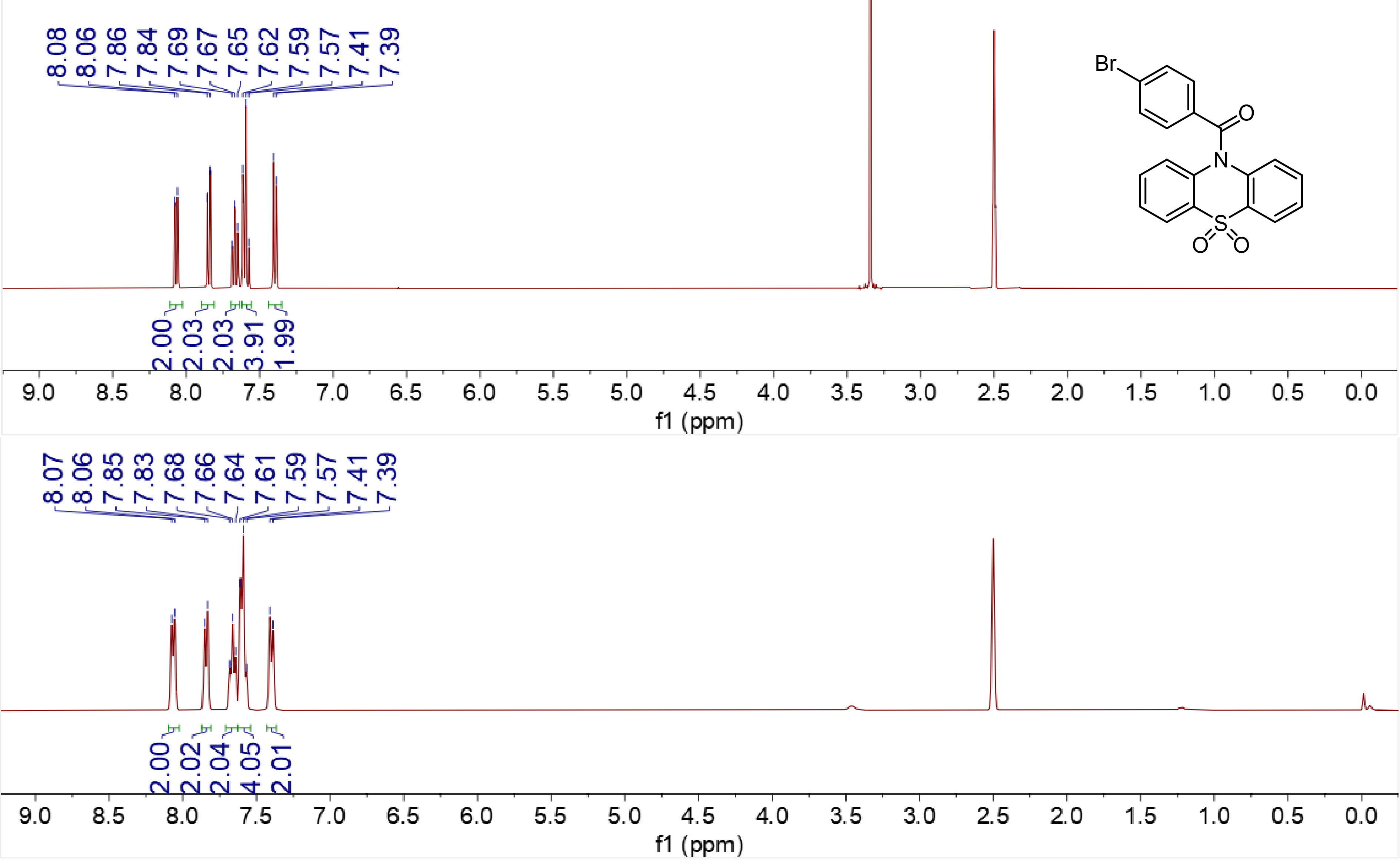


**Figure S5.** ^1^H NMR spectra of *p*BDO molecule before (top) and after (bottom) UV-light irradiation in DMSO-*d*_6_.


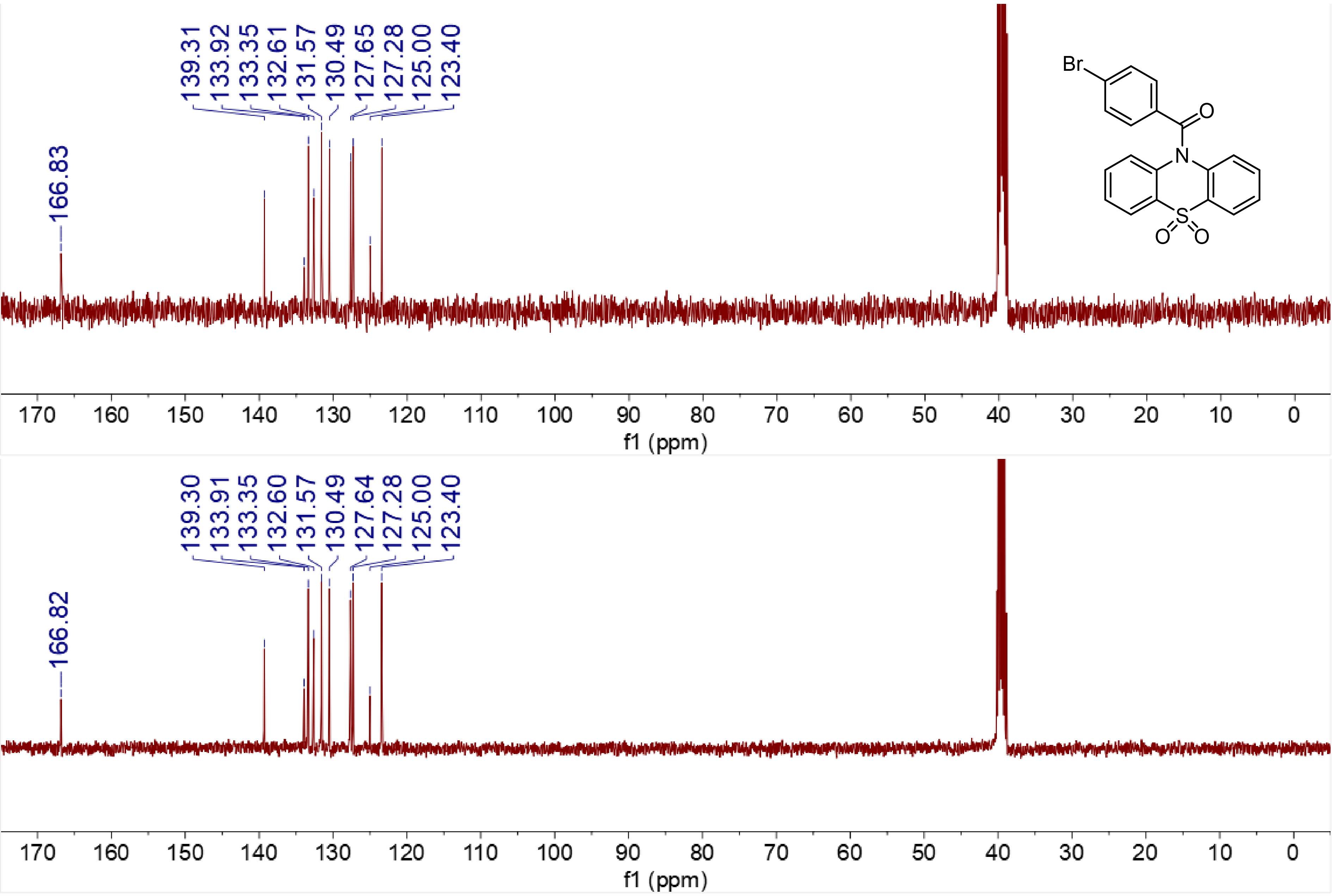


**Figure S6.** ^13^C NMR spectra of *p*BDO molecule before (top) and after (bottom) UV-light irradiation in DMSO-*d*_6_.


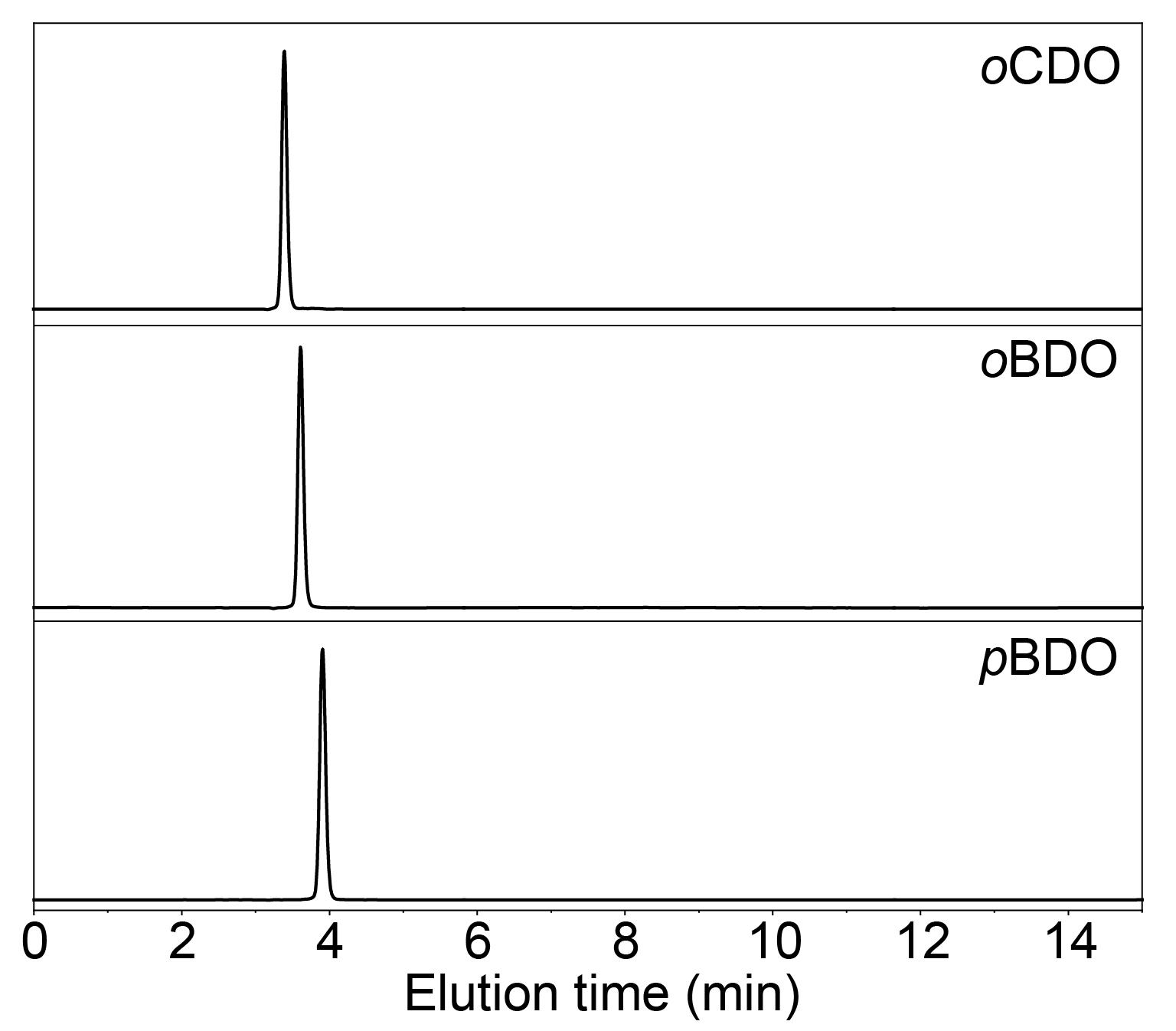


**Figure S7.** High performance liquid chromatography spectra of *o*CDO, *o*BDO and *p*BDO molecules.


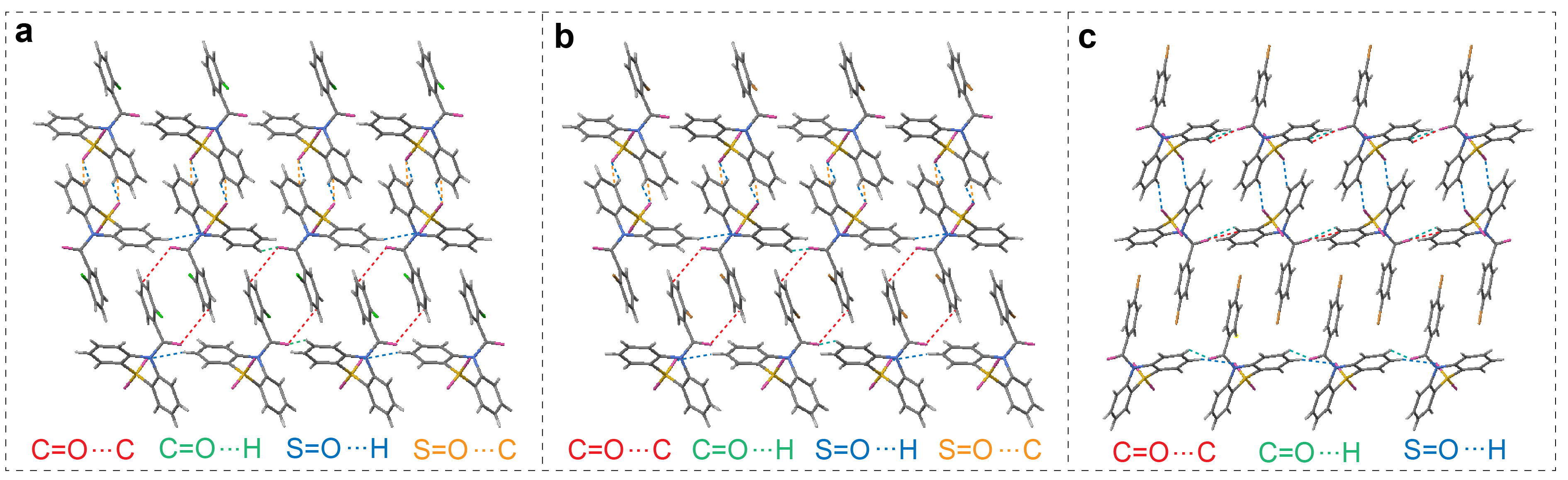


**Figure S8**. Molecular stacking and intermolecular interactions in *o*CDO (a), *o*BDO (b) and *p*BDO (c) crystals.

II. Additional photophysical properties of photo-responsive organic phosphors


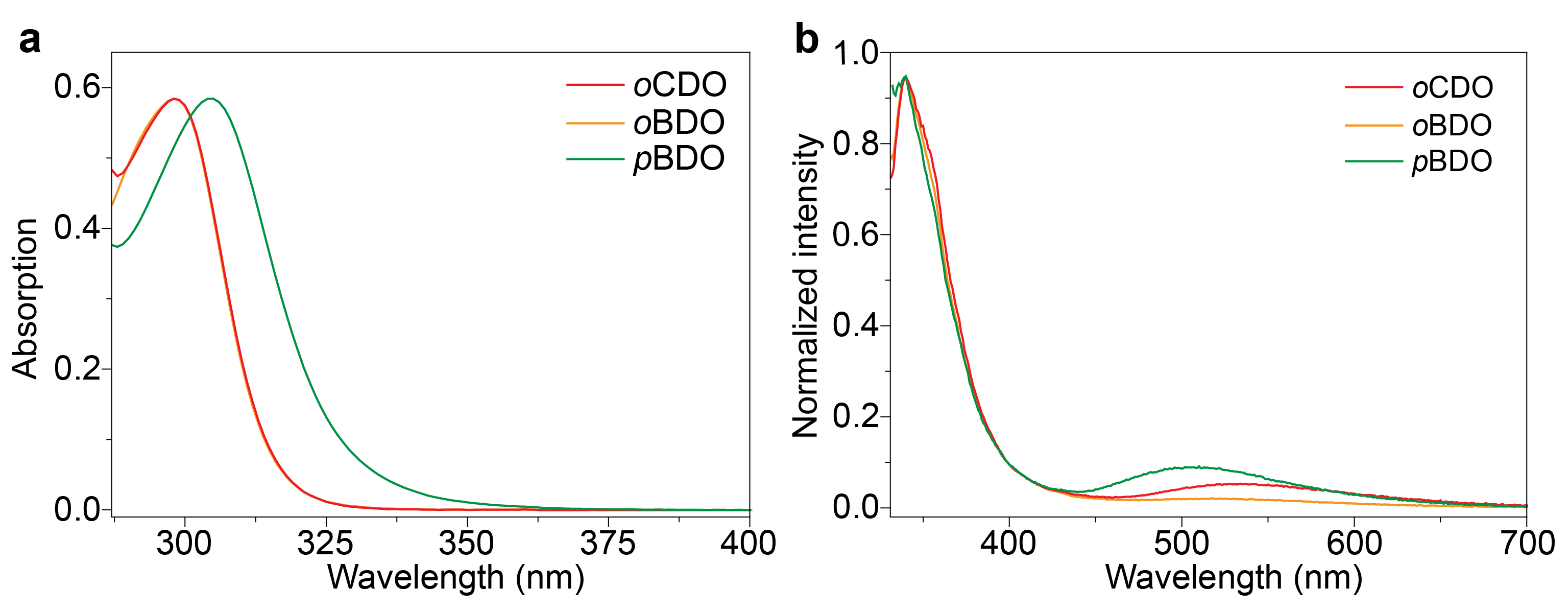


**Figure S9**. Photophysical properties of *o*CDO, *o*BDO and *p*BDO molecules in toluene solution (1×10^-5^ mol·L^-1^). (a) UV absorption spectra. (b) Steady-state photoluminescence (PL) spectra excited by 290 nm under ambient conditions.


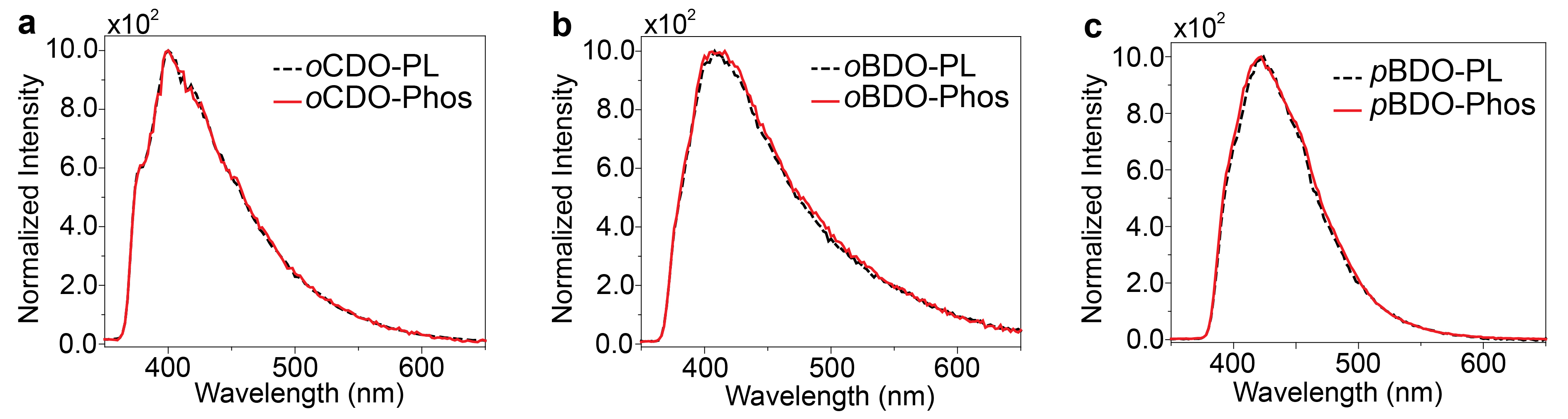


**Figure S10**. Steady-state PL (dotted lines) and phosphorescence spectra (solid lines) for *o*CDO (a), *o*BDO (b) and *p*BDO (c) molecules in dilute toluene solution (1×10^-5^ mol·L^-1^) excited by 290 nm at 77 K.


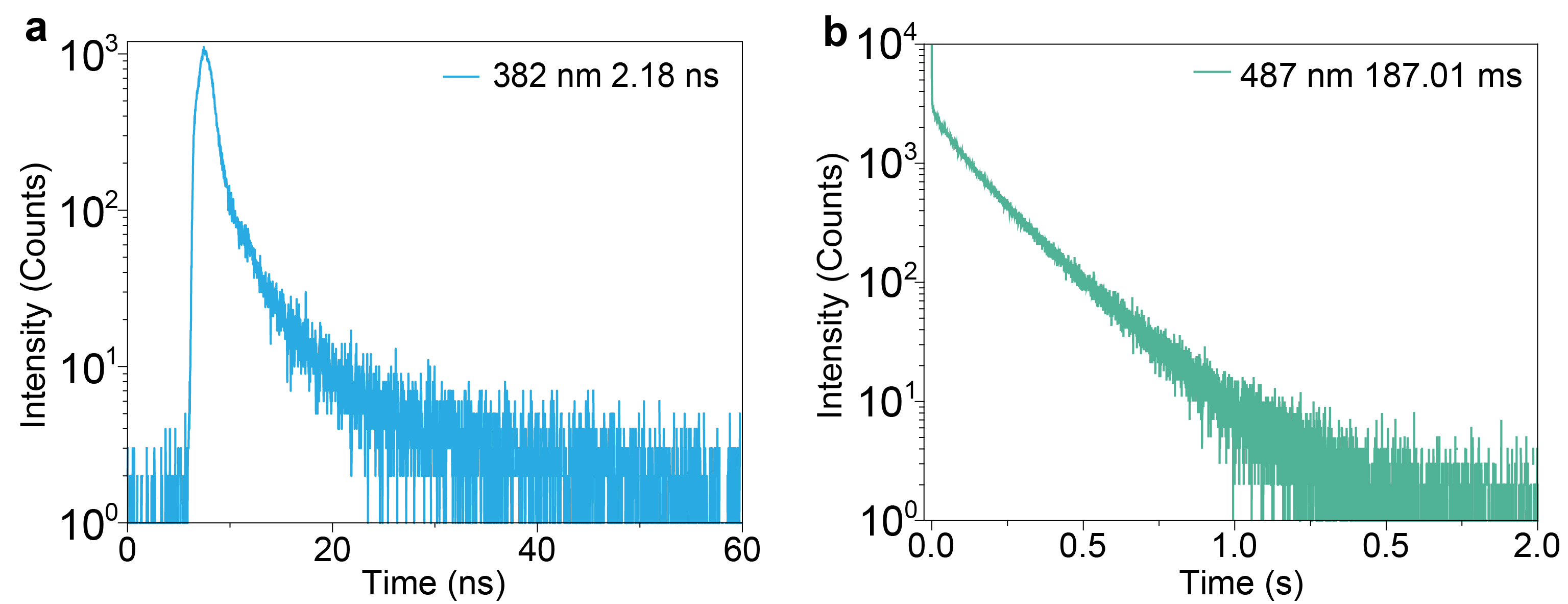


**Figure S11.** Time-resolved decay cures of *o*BDO solid monitoring at 382 (a) and 487 nm (b) under ambient conditions.


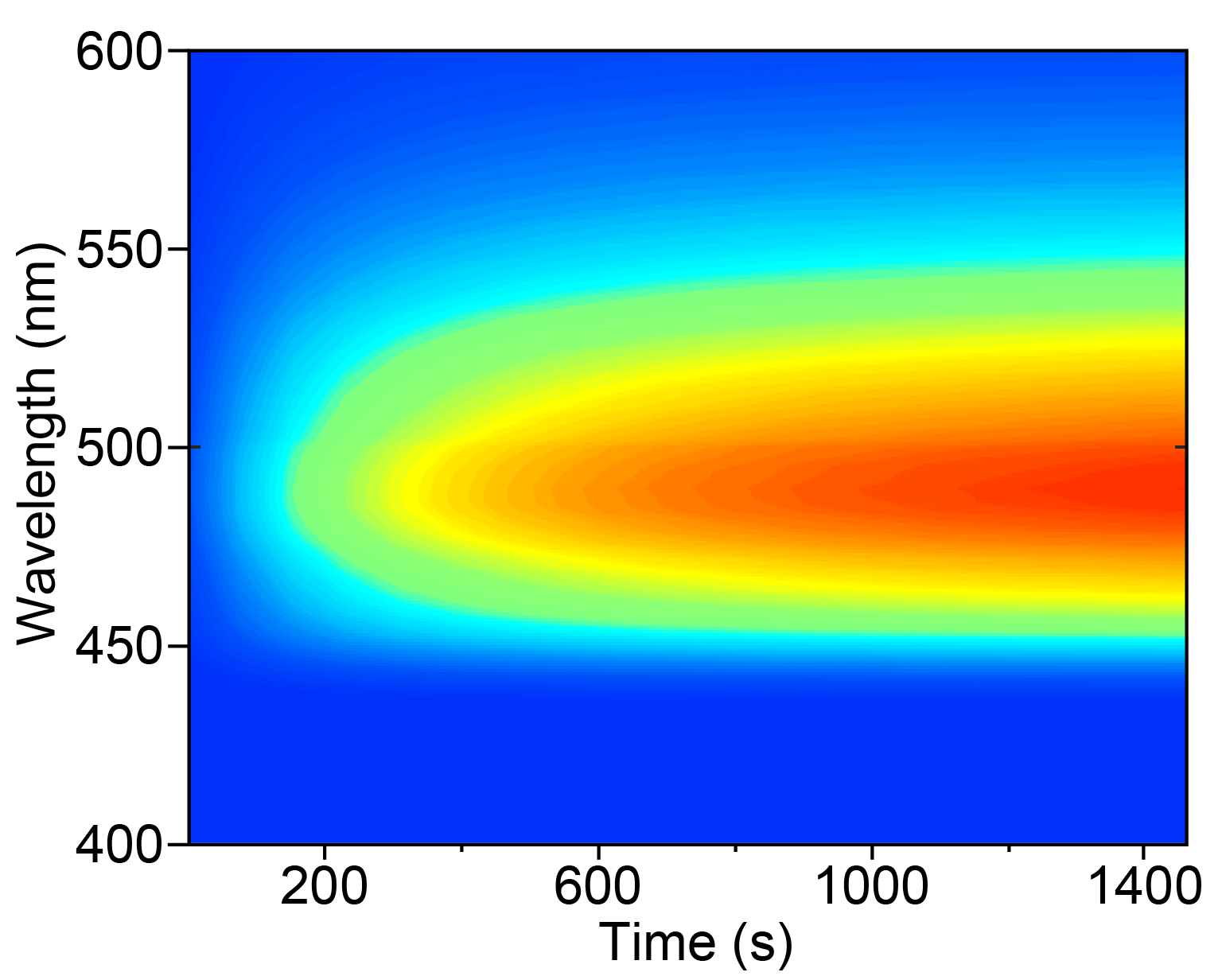


**Figure S12.** Time dependent phosphorescence spectra of *o*BDO solid irradiated by a 330 nm light source with a power density of 284 μW·cm^-2^ under ambient conditions.


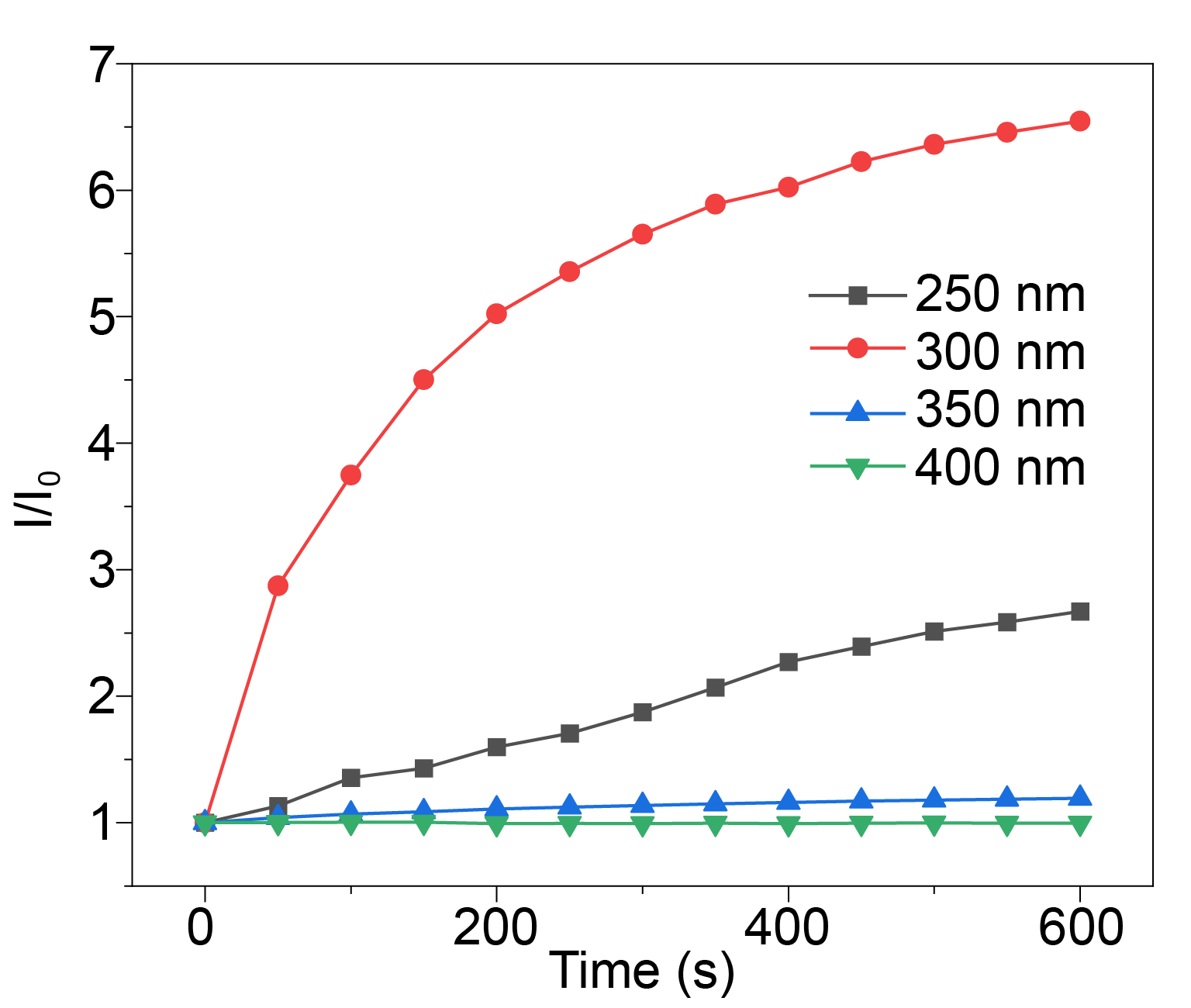


**Figure S13.** Excitation wavelength dependent phosphorescent intensity variation of emission at 489 nm of *o*BDO solid under ambient conditions.


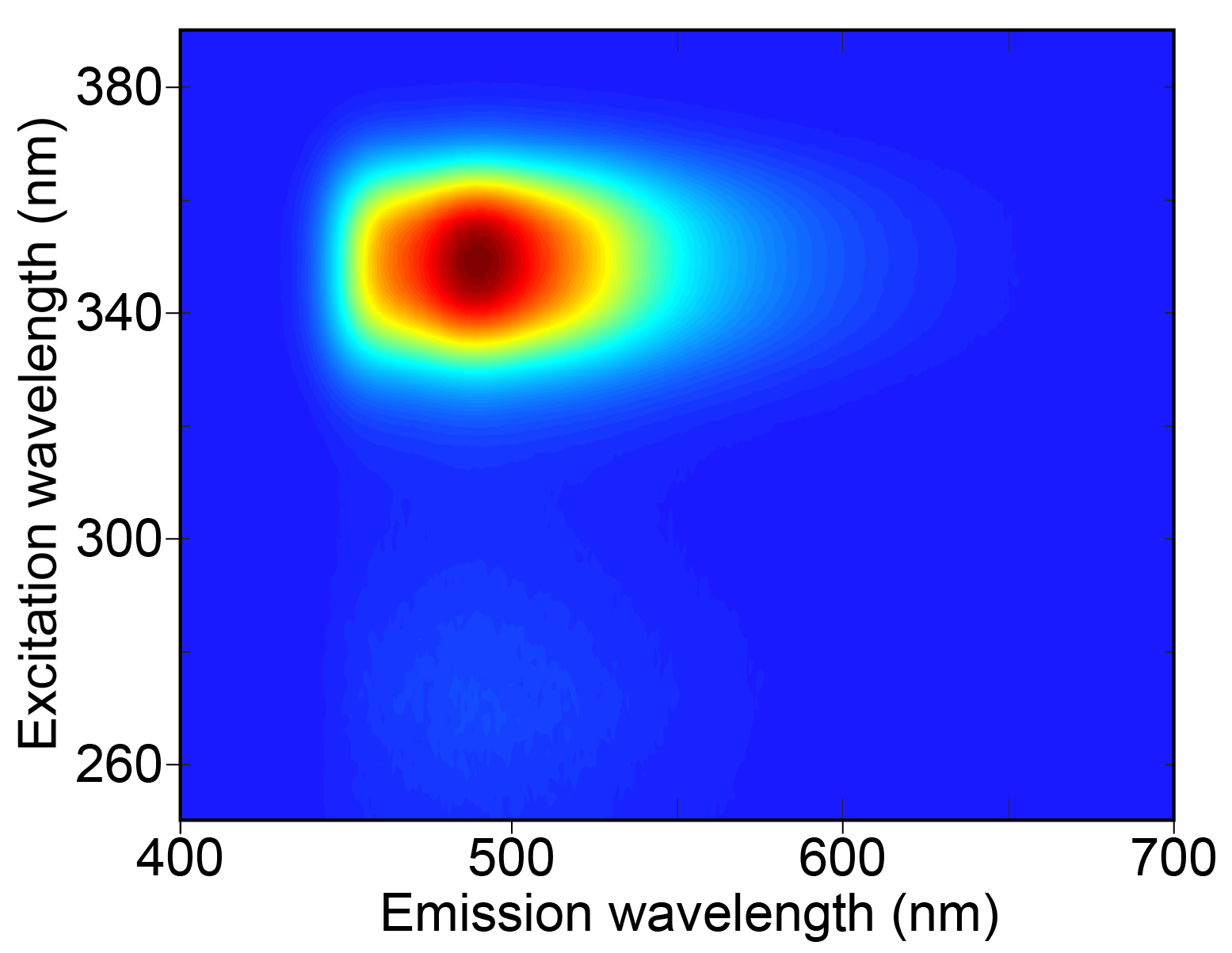


**Figure S14.** Excitation-phosphorescence-emission mapping of *o*BDO solid under ambient conditions.


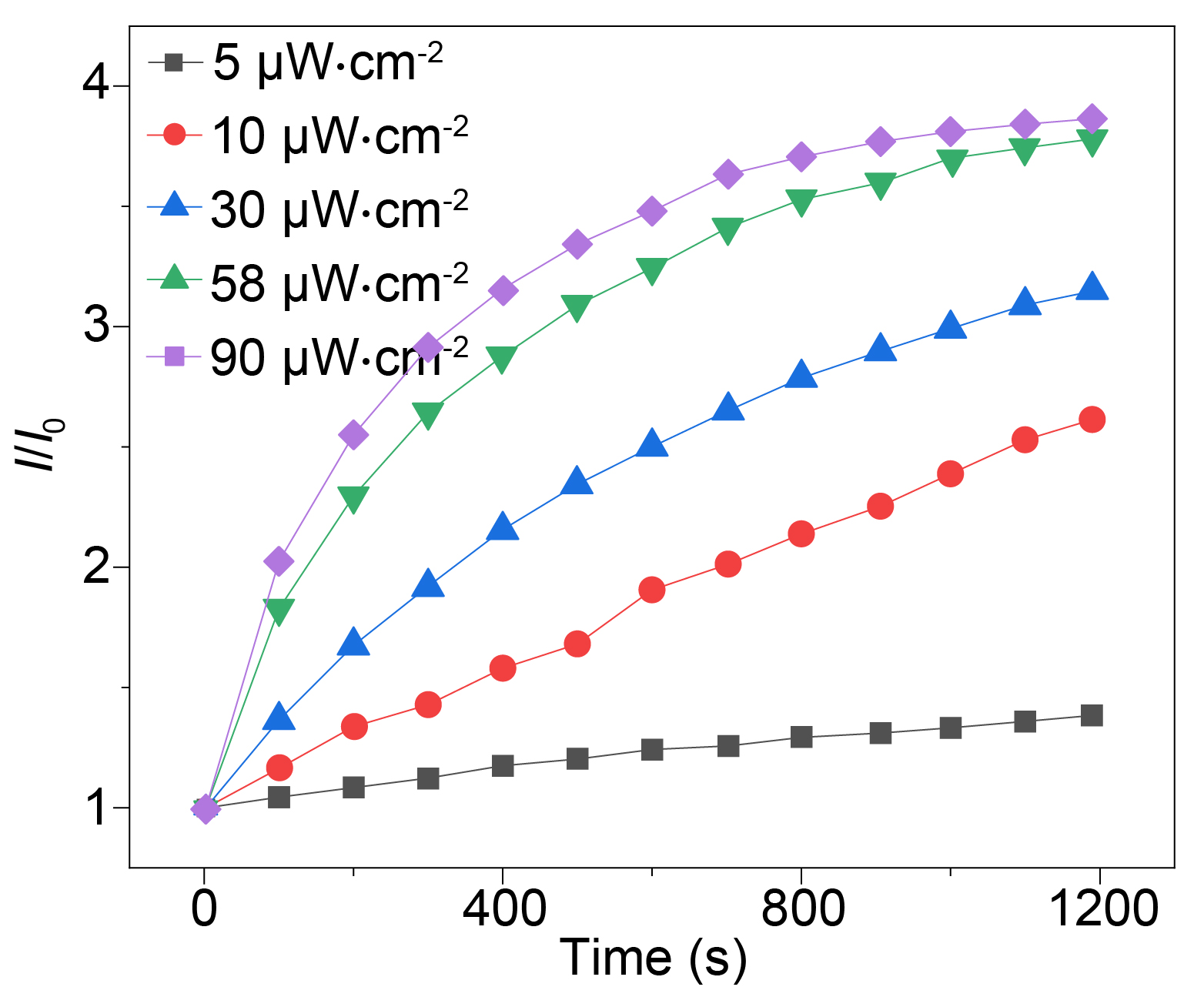


**Figure S15**. Excitation time dependent phosphorescent intensity at 489 nm of *o*BDO solid under excitation of 330 nm with varied power density from 5 to 90 μW·cm^-2^ under ambient conditions.


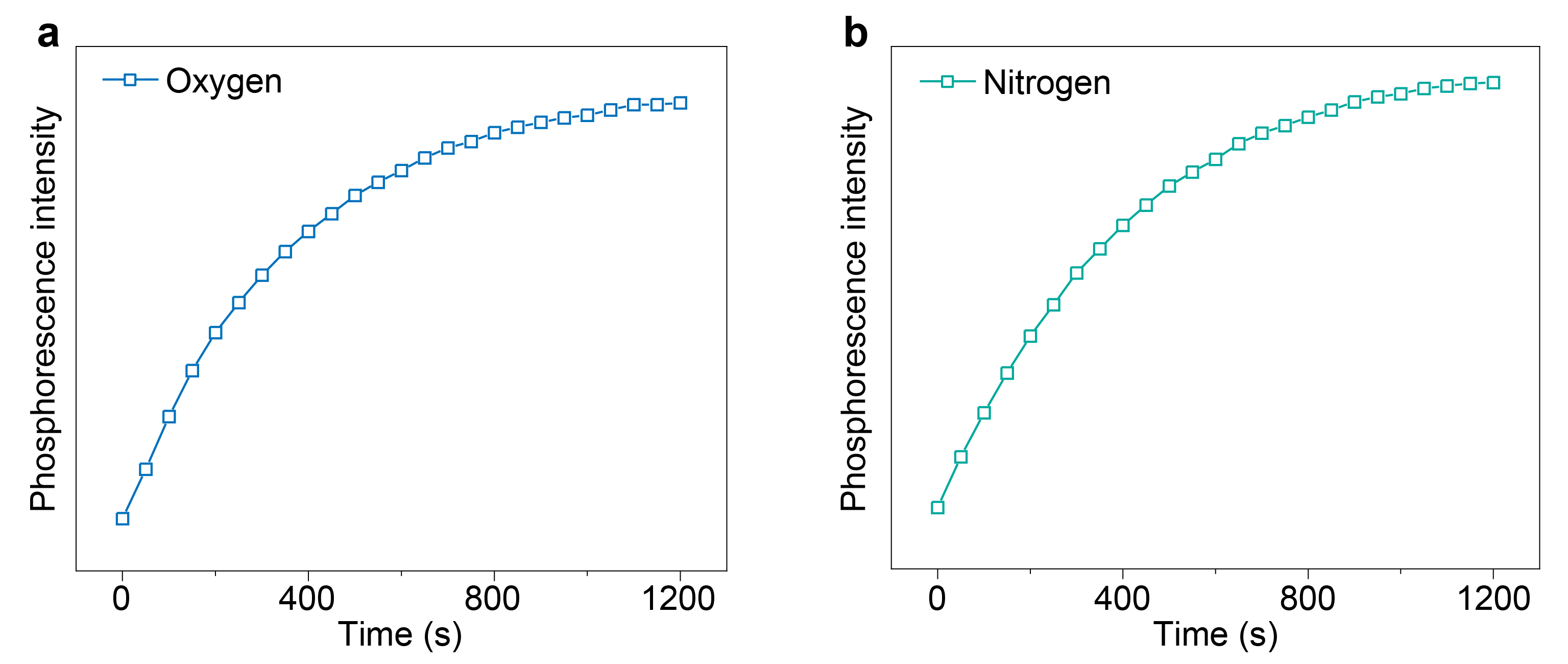


**Figure S16**. Excitation time-dependent phosphorescent intensity of the *o*BDO solid in oxygen (a) and nitrogen (b). Note that the irradiation source is 330 nm UV light with a power density of 284 μW·cm^-2^.


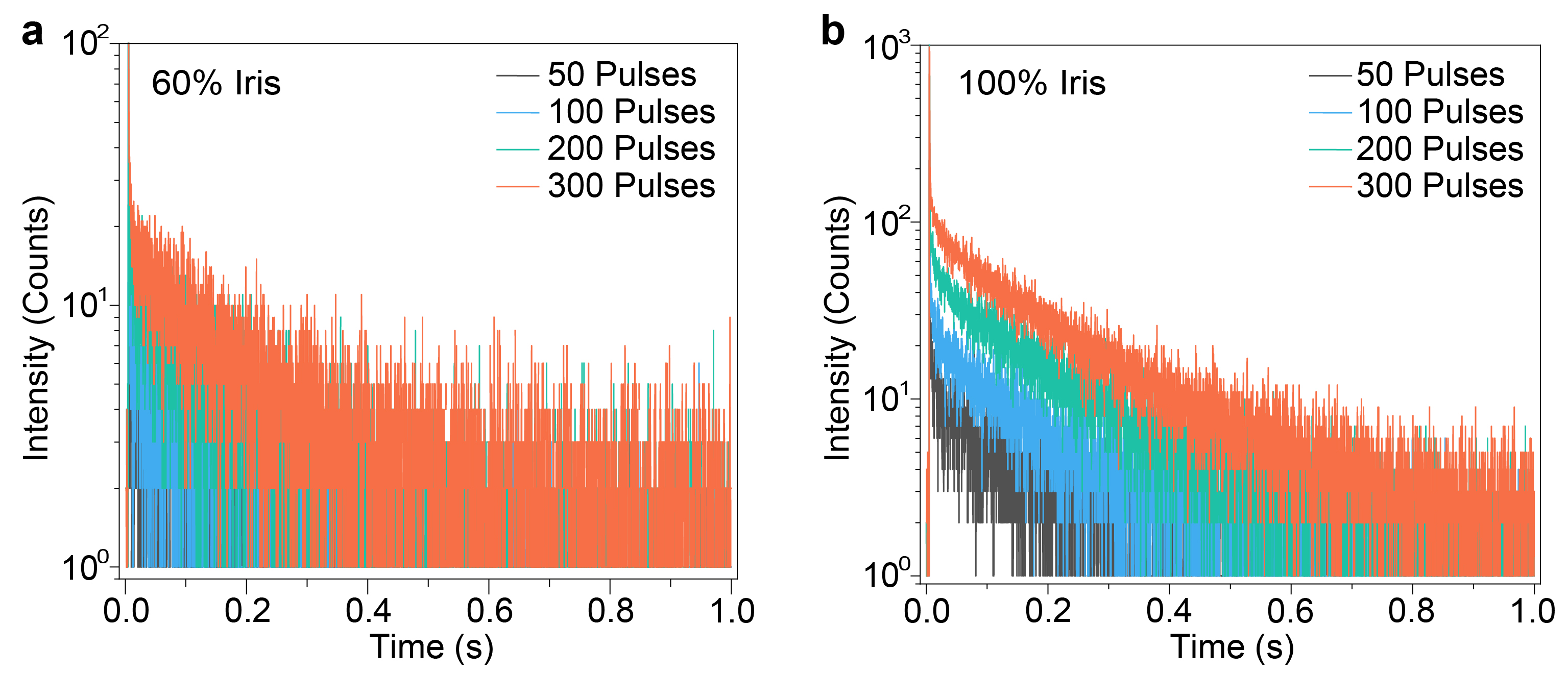


**Figure S17**. Phosphorescent lifetime decay curves of the *o*BDO solid monitoring at 487 nm by regulating different pulse numbers with the light flux of 60% (a) and 100% (b) iris.


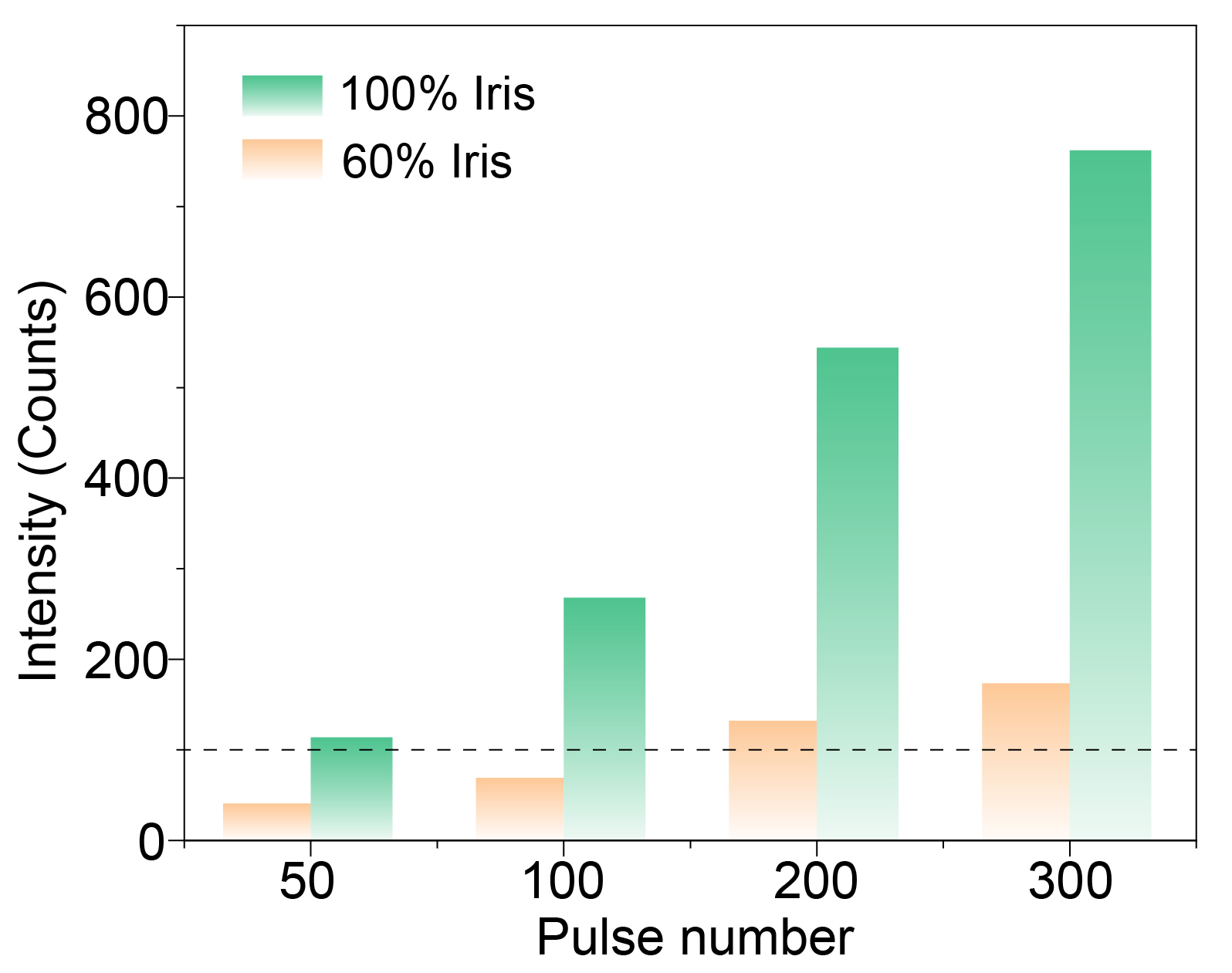


**Figure S18**. Phosphorescent intensity of the *o*BDO solid under different pulse intensity and numbers.

**Table S1.** The phosphorescent intensity and lifetimes of the *o*BDO solid under different pulse intensity and numbers.

| Light flux  (iris) | Pulse number | Luminescent intensity  (Counts) | Luminescent lifetime  (ms) |
| --- | --- | --- | --- |
| 60% | 50 | 40 | 42.21 |
|  | 100 | 68 | 53.97 |
|  | 200 | 131 | 87.12 |
|  | 300 | 174 | 117.42 |
| 100% | 50 | 113 | 72.42 |
|  | 100 | 267 | 114.51 |
|  | 200 | 543 | 142.74 |
|  | 300 | 762 | 163.78 |

III. PAP mechanism investigation and molecular expansion


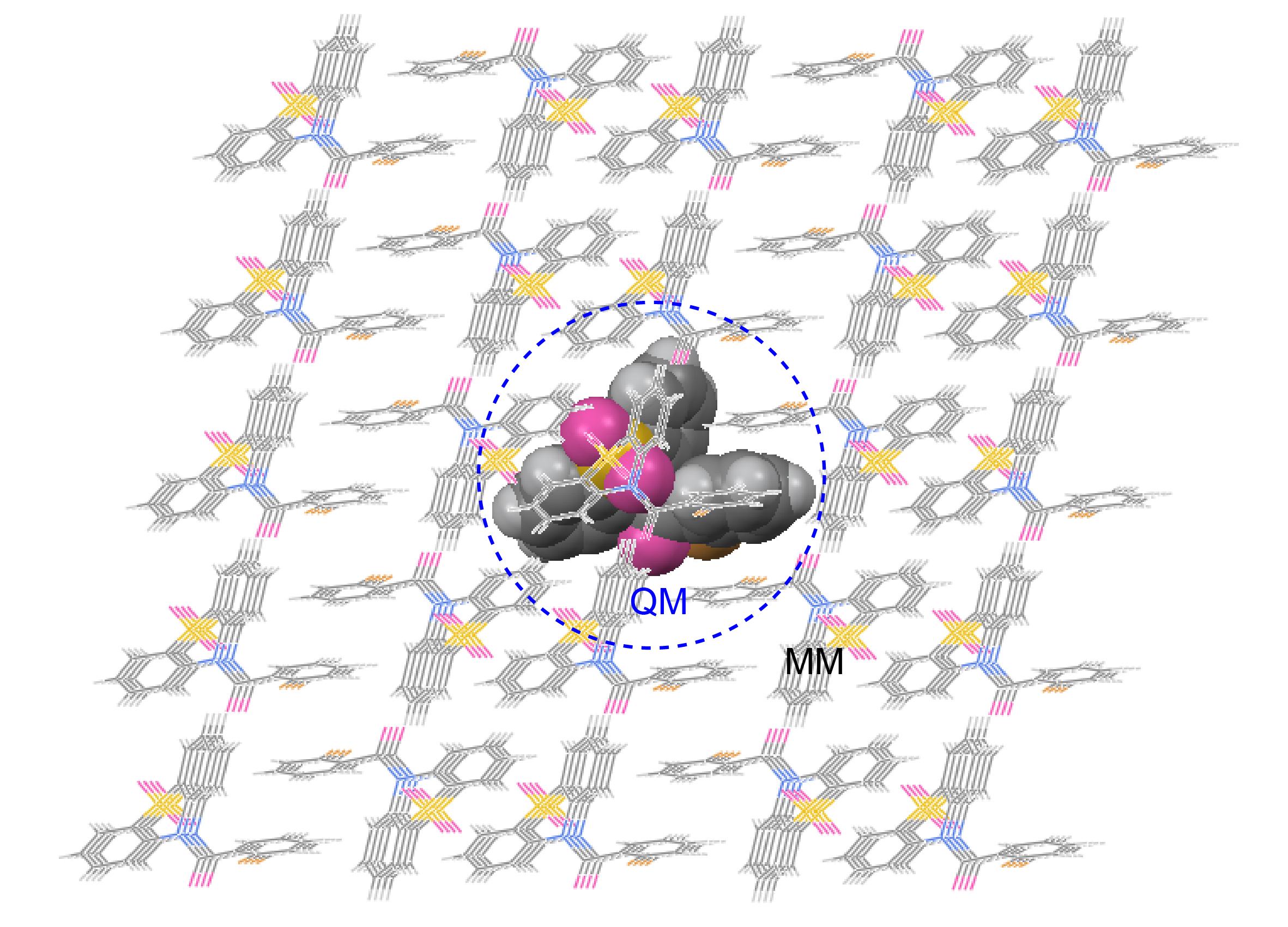


Figure S19. QM/MM modelling of the *o*BDO molecules in the single crystal.


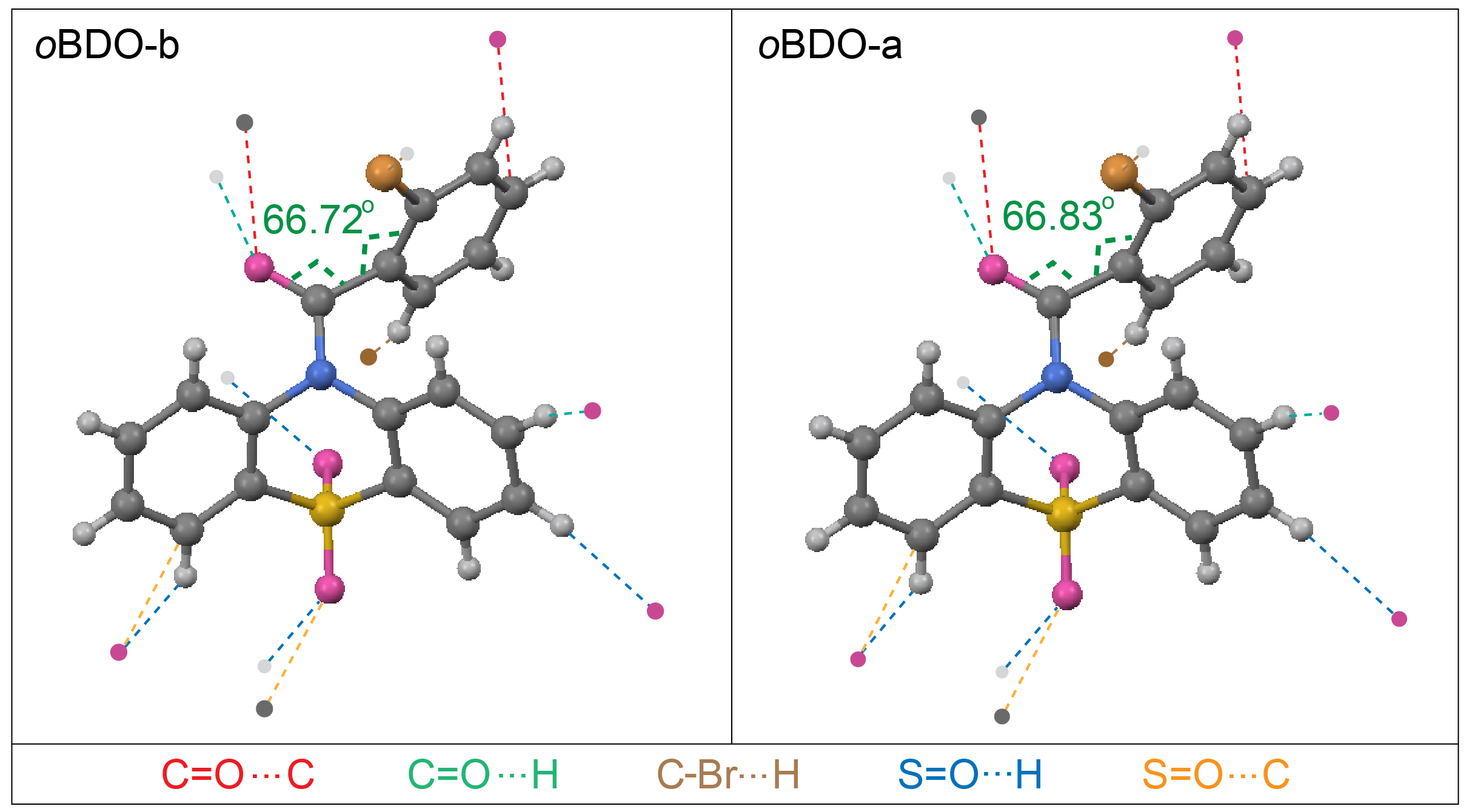


**Figure S20**. Intermolecular interactions and molecular torsion angles between carbonyl group and benzene ring of the *o*BDO before (*o*BDO-b) and after (*o*BDO-a) photoactivation.

**Table S2.** Intermolecular interactions of the *o*BDO before and after photoactivation.

| Interaction type | *o*BDO-b | *o*BDO-a |
| --- | --- | --- |
| C=O···H | 2.493 | 2.486 |
| S=O···H | 2.376 | 2.377 |
|  | 2.591 | 2.588 |
| S=O···C | 3.203 | 3.206 |
| C=O···C | 3.158 | 3.147 |
| C-Br···H | 3.019 | 3.014 |


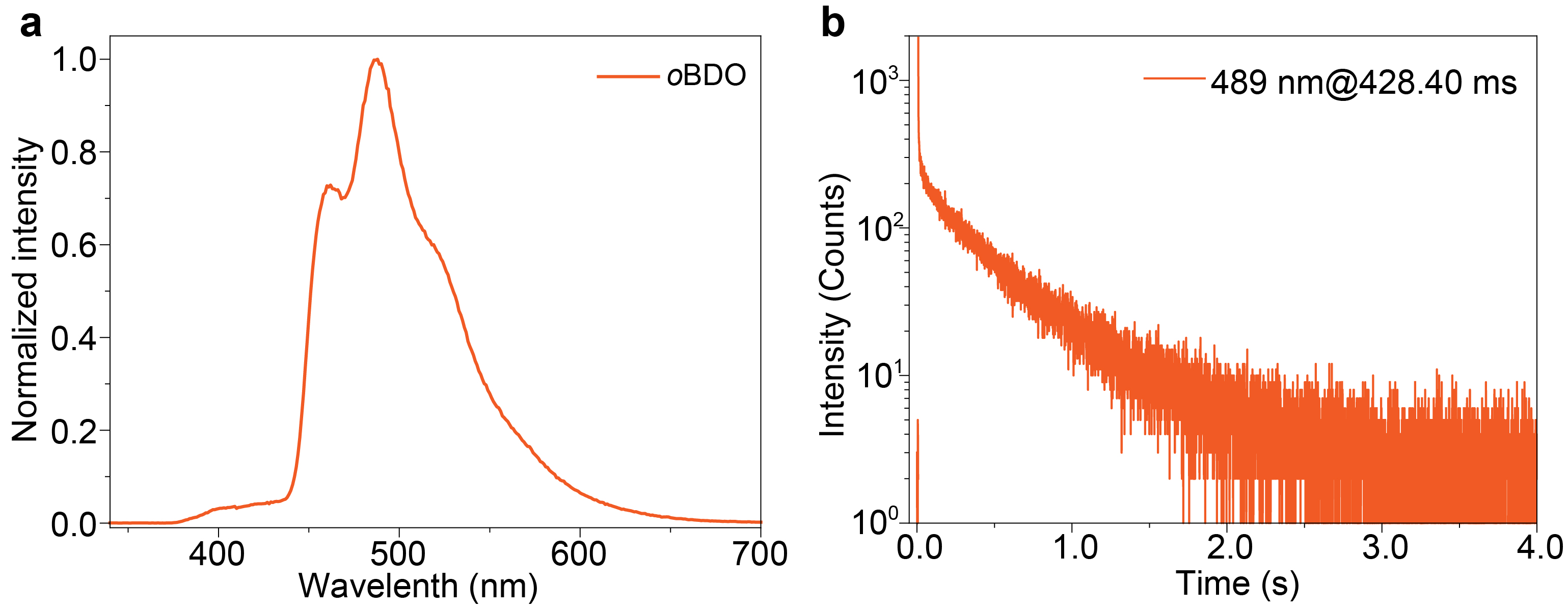


**Figure S21**. Phosphorescence spectrum (a) and phosphorescent lifetime decay curve (b) of *o*BDO solid at 77 K.


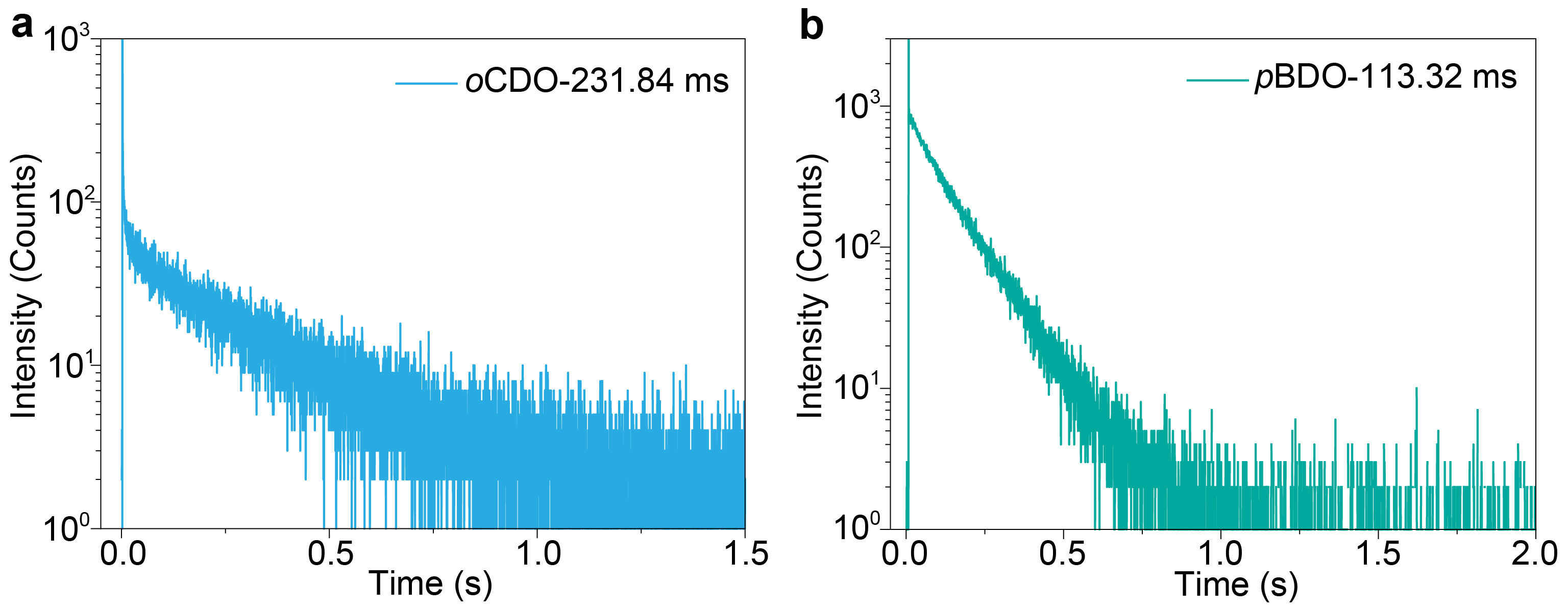


**Figure S22**. Phosphorescent lifetime decay curves of *o*CDO (a) and *p*BDO solids (b) under ambient conditions.


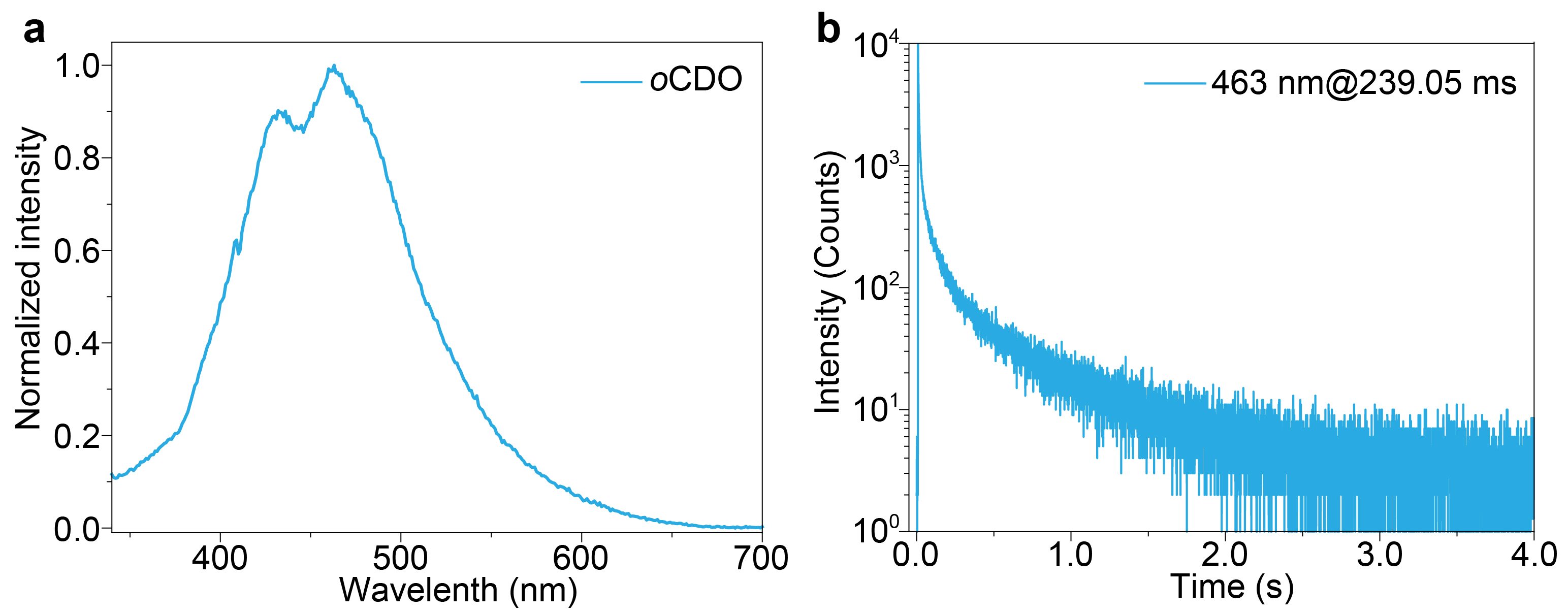


**Figure S23**. Phosphorescence spectrum (a) and phosphorescent lifetime decay curve (b) of *o*CDO solid at 77 K.


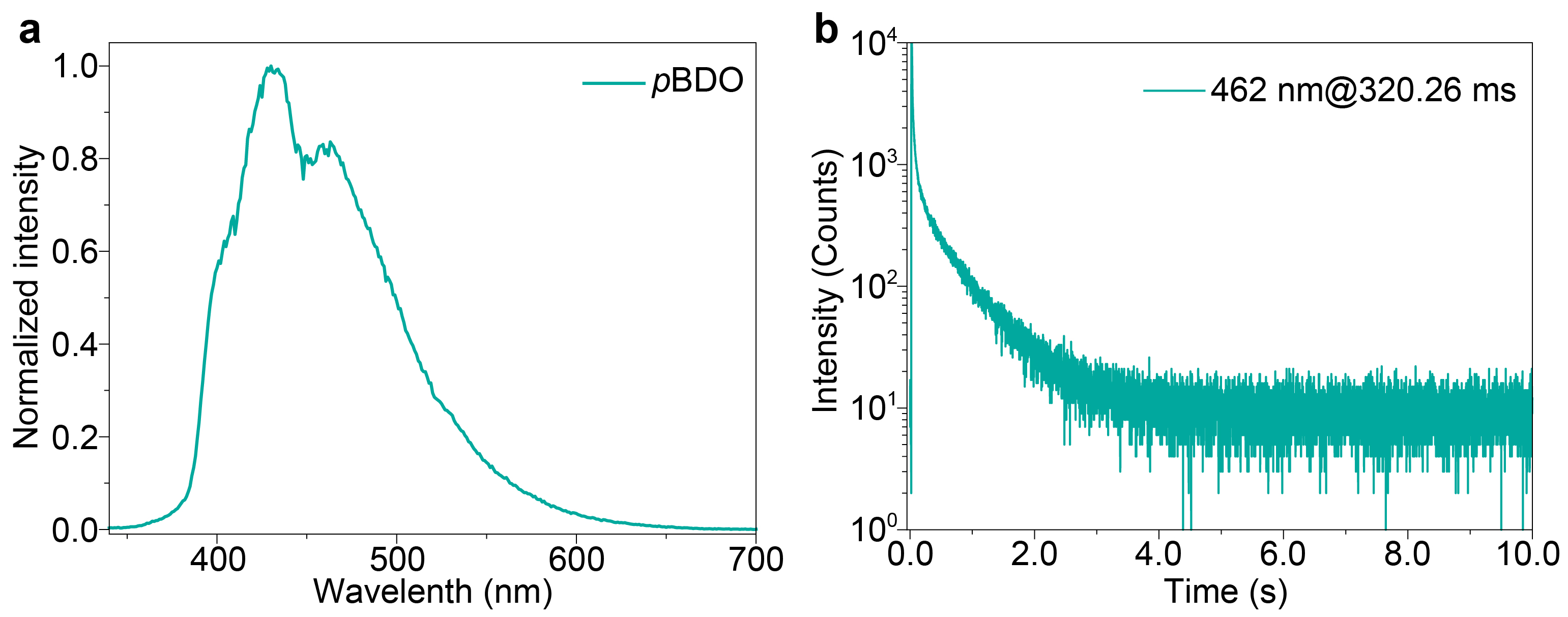


**Figure S24**. Phosphorescence spectrum (a) and phosphorescent lifetime decay curve (b) of *p*BDO solid at 77 K.


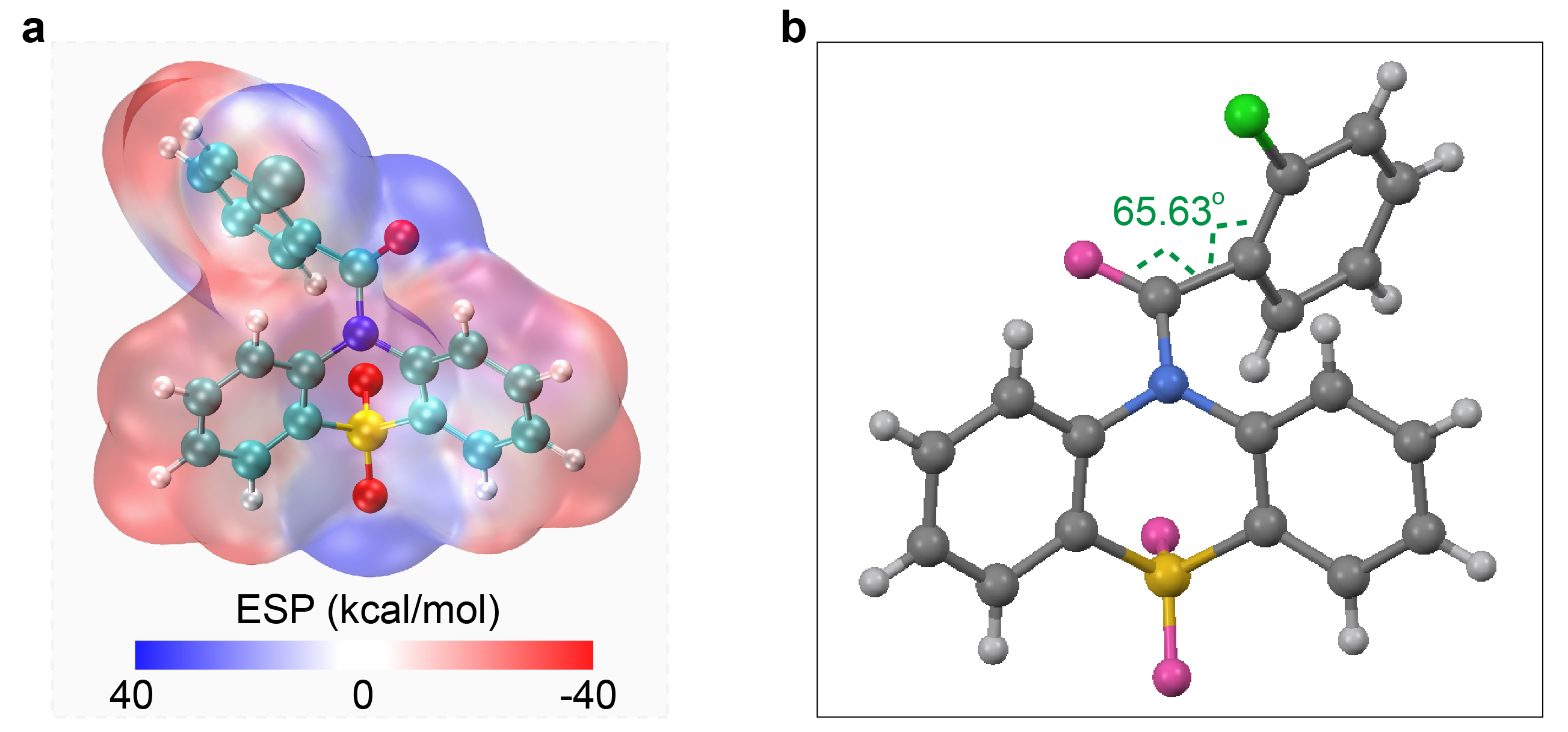


**Figure S25**. Molecular electrostatic potential and molecular configuration in single crystal. (a) Electrostatic potential (ESP) distribution for the *o*CDO molecule in single molecular state. (b) Molecular torsion angle between carbonyl group and benzene ring of *o*CDO molecule in crystal.

**Table S3.** Crystal data of *o*BDO before and after photoactivation at room temperature.

| Name | *o*BDO-b | *o*BDO-a |
| --- | --- | --- |
| Formula | C_19_H_12_BrNO_3_S | C_19_H_12_BrNO_3_S |
| Space Group | P 2_1_/c | P 2_1_/c |
| Cell Lengths (Å) | a=16.064(2)  b=7.7209(9)  c=14.6456(18) | a=16.0628(7)  b=7.7269(4)  c=14.6455(7) |
| Cell Angles (o) | α=90  β=106.007  γ=90 | α=90  β=105.9700(10)  γ=90 |
| Cell Volume (Å^3^) | 1746.04 | 1747.58 |
| Z, Z' | Z: 4; Z': 0 | Z: 4; Z': 0 |
| Density (g·cm^-3^) | 1.576 | 1.575 |
| CCDC number | 2169586 | 2169583 |

**Table S4.** Crystal data of *o*CDO and *p*BDO at room temperature.

| Name | *o*CDO | *p*BDO |
| --- | --- | --- |
| Formula | C_19_H_12_ClNO_3_S | C_19_H_12_BrNO_3_S |
| Space Group | P 2_1_/c | P -1 |
| Cell Lengths (Å) | a=16.260(8)  b=7.472(3)  c=14.733(8) | a=6.5909(6)  b=7.8573(8)  c=17.5378(16) |
| Cell Angles (o) | α=90  β=106.070(16)  γ=90 | α=95.706(3)  β=95.231(3)  γ=104.178(3) |
| Cell Volume (Å^3^) | 1720.04 | 869.892 |
| Z, Z' | Z: 4; Z': 0 | Z: 2; Z': 0 |
| Density (g·cm^-3^) | 1.428 | 1.582 |
| CCDC number | 2169585 | 2169584 |

Ⅳ. Supplementary video

The supplementary videos were recorded in the dark with a camera (Canon EOS 700D).

**SV.** From the video, *o*BDO crystal exhibited fleeting and hard to observed phosphorescence after irradiation by UV light for 2 seconds. After continuous irradiation for 2 minutes, green phosphorescence visible to the naked eye was captured and can last for about 3 seconds, demonstrating remarkable photo-activated dynamic phosphorescent behavior.
