# Supplementary material for: Photoactivated organic phosphorescence by stereo-hindrance engineering for mimicking synaptic plasticity
Source: Light Sci Appl. 2023 Apr 10;12:90. doi: 10.1038/s41377-023-01132-3 (PMC10086021; doi:10.1038/s41377-023-01132-3)
Supplement: Supplementary file 3 — Authorship Change Form [file 41377_2023_1132_MOESM3_ESM.pdf]

Authorship Change Form

Any changes to the author list after submission, such as a change in the order of the authors, or the deletion or addition of authors, must be approved by a signed letter from every author.

Date: January 31, 2023

Journal Name: Light Science & Applications

Manuscript Number: LSA20221968

Manuscript Title: Photoactivated organic phosphorescence by stereo-hindrance engineering for mimicking synaptic plasticity

Reason for authorship change:

Dr. Xiao Wang contributed significantly to the revision of the manuscript.

Prof. Jun Yin played an important role in discussion of the test results.

Previous Author List:

He Wang, Yuan Zhang, Chifeng Zhou, Huili Ma, Huifang Shi, Zhongfu An and Wei Huang

Updated Author List (the changes have been distinguished from original list using yellow color):

He Wang, Yuan Zhang, Chifeng Zhou, Xiao Wang, Huili Ma, Jun Yin, Huifang Shi, Zhongfu An and Wei Huang

We, the undersigned authors, agree to the authorship changes detailed above for the manuscript listed at the top of this form:

Author name: He Wang **Author name:** He Wang

Author name: Yuan Zhang **Author name:** Yuan Zhang

Author name: Chifeng Zhou **Author name:** Chifeng Zhou

Author name: Huili Ma **Author name:** MA Huili

Author name: Huifang Shi **Author name:** Huifang Shi

Author name: Zhongfu An **Author name:** [illegible]

Author name: Wei Huang **Author name:** [illegible]
